# Synthesis of Dihydrouracils Spiro-Fused to Pyrrolidines: Druglike Molecules Based on the 2-Arylethyl Amine Scaffold

**DOI:** 10.3390/molecules15042269

**Published:** 2010-03-30

**Authors:** Daniel Blanco-Ania, Carolina Valderas-Cortina, Pedro H.H. Hermkens, Leo A.J.M. Sliedregt, Hans W. Scheeren, Floris P.J.T. Rutjes

**Affiliations:** 1 Institute for Molecules and Materials, Radboud University Nijmegen, Heyendaalseweg 135, 6525 AJ Nijmegen, The Netherlands; 2 MSD Research Laboratories, P.O. Box 20, 5340 BH Oss, The Netherlands; 3 Solvay Pharmaceuticals, Sector Discovery Weesp, P.O. Box 900, 1380 DA Weesp, The Netherlands

**Keywords:** Knoevenagel condensation, 1,3-dipolar cycloaddition, azomethine ylide, parallel synthesis, spiro dihydrouracils

## Abstract

The synthesis of a small library of dihydrouracils spiro-fused to pyrrolidines is described. These compounds are synthesized from β-aryl pyrrolidines, providing products with the *2-arylethyl amine* moiety, a structural feature often encountered in compounds active in the central nervous system. The β-aryl pyrrolidines are synthesized through a three-step methodology that includes a Knoevenagel condensation reaction, a 1,3-dipolar cycloaddition reaction, and a nitrile reduction.

## 1. Introduction

**Figure 1:**
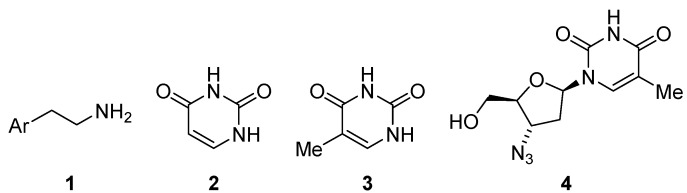
2-Arylethyl amine (**1**), uracil (**2**), thymine (**3**), and zidovudine (AZT, **4**).

The *2-arylethyl amine* moiety **1** (Figure 1) is an important privileged structure, which is encountered in numerous compounds active in the central nervous system (CNS). This privileged structure is present in neurotransmitters such as dopamine, epinephrine, norepinephrine, and serotonin [1]. Salmeterol [2,3] and venlafaxine [4,5,6], two of the ten best-selling prescription drugs in 2006 [7], also contain this moiety. In addition, the 2-arylethyl amine unit occurs in many hallucinogenic drugs, such as LSD, MDMA (ecstasy), mescaline, and psilocybin (magic mushrooms) [8,9].

On the other hand, pyrimidine-2,4-diones are a class of bioactive heterocyclic molecules, the most famous examples being uracil (**2**) and thymine (**3**, Figure 1), which form part of the nucleotides of RNA and DNA, respectively [10]. Pyrimidine-2,4-diones have attracted considerable attention in the pharmaceutical industry as anti-inflammatory agents [11], dopamine receptor agonists [12], serotonin uptake inhibitors [13], and antiepileptic agents [14]. A good example of a pyrimidine-2,4-dione derivative with a medicinal application is zidovudine (AZT, **4**, Figure 1), used as an anti-AIDS agent [15]. This moiety is also the core structural element of some fungicides [16] and herbicides [17].

**Scheme 1:**
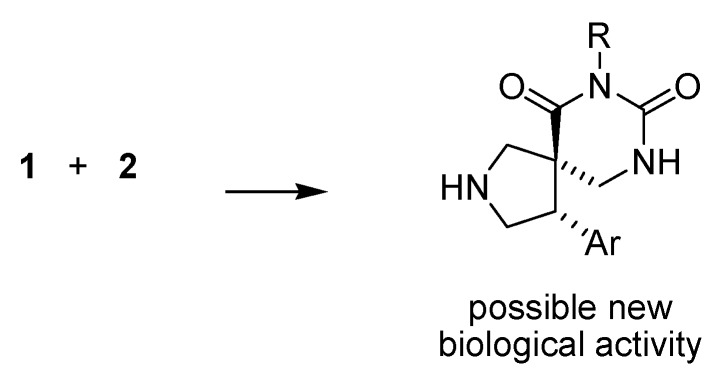
Combination of two privileged structures to generate a product with increased rigidity.

It is known that the difference in activity and receptor selectivity of drugs might be explained by the conformation of the contained privileged structure. Generation of semi-rigid drugs facilitates the study of their interactions with the receptors, may lead to more selective interactions with fewer side effects, and permits the rational design of more potent and selective drugs in the future [18,19]. Herein we present the parallel synthesis of a library comprised of compounds combining all the above features (Scheme 1). First, these compounds possess a β-aryl pyrrolidine with a conformationally constrained 2-arylethyl amine. Second, they are semirigid structures because of the spiro fusion to a dihydrouracil. It is known that the combination of privileged structures can lead to new chemical entities that may have pharmacological relevance [20,21] and increase the structural diversity.

## 2. Results and Discussion

**Scheme 2:**
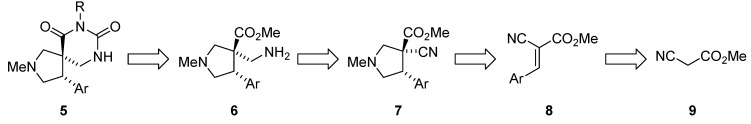
Retrosynthetic analysis for the synthesis of spiro dihydrouracils **5**.

We envisioned that a suitable strategy to synthesize these compounds may proceed as shown retrosynthetically in Scheme 2. The spiro dihydrouracils **5** could be synthesized by an annulation reaction of α-aminomethyl esters **6** and an isocyanate. The aminomethyl group of compounds **6** could be derived from a masked amino function, such as a cyano group, by reduction. Compounds **7** possess two electron-withdrawing groups at the carbon in the 3-position of the pyrrolidine, rendering it a perfect pattern for preparation by a 1,3-cycloaddition reaction of an azomethine ylide and an electron-deficient alkene. This would leave the 3-aryl-2-cyanoacrylates **8** as starting materials, which could be obtained by the Knoevenagel condensation reaction of methyl 2-cyanoacetate (**9**) and an aromatic aldehyde.

### 2.1. Knoevenagel condensation reaction

**Figure 2:**
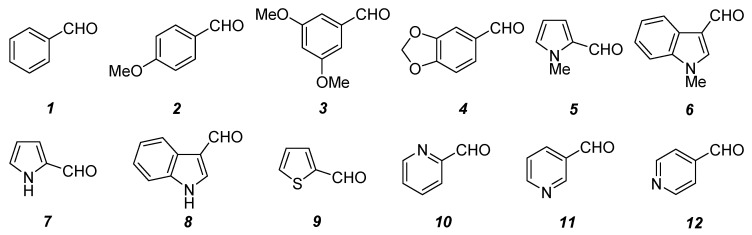
Aromatic aldehydes **10**{*1*–*12*} used for the Knoevenagel reaction.

The synthesis of compound class **5** commenced with the condensation reaction of methyl 2-cyanoacetate (**9**) and aromatic aldehydes **10**. Twelve aldehydes **10**{*1*–*12*} (electron-rich aromatic, electron-rich heteroaromatic, and electron-poor heteroaromatic aldehydes; Figure 2) were selected for the formation of the scaffolds.

The reaction conditions for the Knoevenagel condensation reaction are critically dependent on the electron-withdrawing groups bound to the activated methylene [22] and need to be optimized in every case. Initially, these reactions were performed using EtOH as solvent, but transesterification (up to 3%) was observed and the resulting mixture of methyl and ethyl esters was impossible to separate. These reactions also took place in THF (see entry 8, Table 1), but required a longer reaction time. Finally, treatment of **9** with a catalytic amount of piperidine in MeOH (except for entry 8 because of the low solubility of **10**{*8*} in MeOH) at room temperature produced the desired acrylates **8**{*1*–*12*} in excellent yields (Table 1). All these compounds are only sparingly soluble in MeOH, allowing the pure crystalline products to be easily collected by filtration. These products can also be recrystallized from MeOH yielding crystals of >99.5% purity. The reaction was completely stereoselective in all cases [23], only the *E* alkenes were observed as could be inferred from the ^13^C-NMR coupling constants between the olefinic proton and the carbon atoms of the ester and the nitrile [24,25]. These values are ^3^*J* = 6.6–6.9 Hz for the carbonyl group and ^3^*J* = 13.6–13.9 Hz for the cyano group. The Knoevenagel adducts are stable at room temperature and unreactive towards the regular atmosphere, therefore remain unchanged for months.

**Table 1:** Knoevenagel condensation reaction to form the 2-cyanoacrylates **8**{*1*–*12*}.

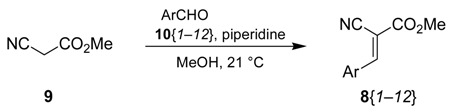
**Entry**	**ArCHO**	**Product**	**Time (min)**	**Yield (%)**
1	**10**{*1*}	**8**{*1*}	30	99
2	**10**{*2*}	**8**{*2*}	30	94
3	**10**{*3*}	**8**{*3*}	40	99
4	**10**{*4*}	**8**{*4*}	60	99
5	**10**{*5*}	**8**{*5*}	120	95
6	**10**{*6*}	**8**{*6*}	120	96
7	**10**{*7*}	**8**{*7*}	30	99
8	**10**{*8*}	**8**{*8*}	480^a^	99
9	**10**{*9*}	**8**{*9*}	30	94
10	**10**{*10*}	**8**{*10*}	30	99
11	**10**{*11*}	**8**{*11*}	90	93
12	**10**{*12*}	**8**{*12*}	25	99

^a^ THF was used as solvent.

### 2.2. 1,3-Dipolar cycloaddition reaction

The next step in the synthesis was the formation of the pyrrolidine-core structures by a 1,3-dipolar cycloaddition reaction using an azomethine ylide. This reaction is an important method for the formation of pyrrolidines [26] and has been used in the synthesis of natural products [27,28]. Among the vast number of procedures for making azomethine ylides [29,30,31,32,33,34,35,36,37,38,39,40,41], the decarboxylative condensation of α-amino acids with aldehydes, typically heated in toluene or DMF, was chosen [42]. Thus, the reaction of paraformaldehyde and sarcosine (*N*-methylglycine) in refluxing toluene in the presence of the 2-cyanoacrylates **8** cleanly provided the desired pyrrolidines **7**, containing the 2-arylethyl amine motif (Table 2). The reaction was clean to such an extent that in some cases an extraction (H_2_O/Et_2_O) was all the purification needed (or just a short column chromatography). The reaction was totally stereospecific in most cases (entries 1–6 and 8), highly stereospecific for **8**{*9*} (entry 9), and partially stereospecific for the electron-poor heteroaryls **8**{*10*-*12*} (entries 10–12) [43]. The mixtures of diastereoisomers that arose could not be separated by column chromatography. The reaction did not take place with compound **8**{*7*} (entry 7); after 4 h only some minor unidentified compounds were formed and most of the substrate was recovered, but there was no trace of compound **7**{*7*}. The reaction of substrate **8**{*5*} did form the product **7**{*5*}, but with a lower yield compared to all the others. The reaction of substrate **8**{*8*} formed the expected cycloadduct, but the indolic nitrogen was (dimethylamino)methylated during the reaction.

**Table 2:** 1,3-Dipolar cycloaddition reaction to form the pyrrolidine-core structures **7**.

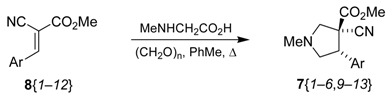
**Entry**	**Acrylate**	**Product(s)^a^**		**Time (min)**	**Yield (%)**
1	**8**{*1*}	**7**{*1*}		20	94
2	**8**{*2*}	**7**{*2*}		75	99
3	**8**{*3*}	**7**{*3*}		80	95
4	**8**{*4*}	**7**{*4*}		80	99
5	**8**{*5*}	**7**{*5*}		120	52
6	**8**{*6*}	**7**{*6*}		80	95
7	**8**{*7*}	**7**{*7*}		240	-
8	**8**{*8*}	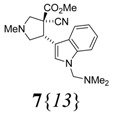		150	85
9	**8**{*9*}	**7**{*9*} +	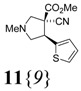	80	87^b^
10	**8**{*10*}	**7**{*10*} +	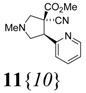	45	90^c^
11	**8**{*11*}	**7**{*11*} +	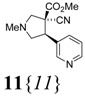	25	72^d^
12	**8**{*12*}	**7**{*12*} +	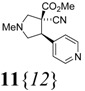	45	85^e^

^a^ Ratio calculated by integration of the ^1^H-NMR signals of the crude reaction mixture; ^b^ **7**{*9*}/**11**{*9*} = 70:1; ^c^ **7**{*10*}/**11**{*10*} = 6.5:1; ^d^ **7**{*11*}/**11**{*11*} = 6.6:1; ^e^ **7**{*12*}/**11**{*12*} = 5:1

After analysis of the results obtained so far, it was decided to continue the research only with the diastereomerically pure compounds **7**{*1*–*6*} for the construction of the library scaffolds.

### 2.3. Reduction

**Figure 3:**
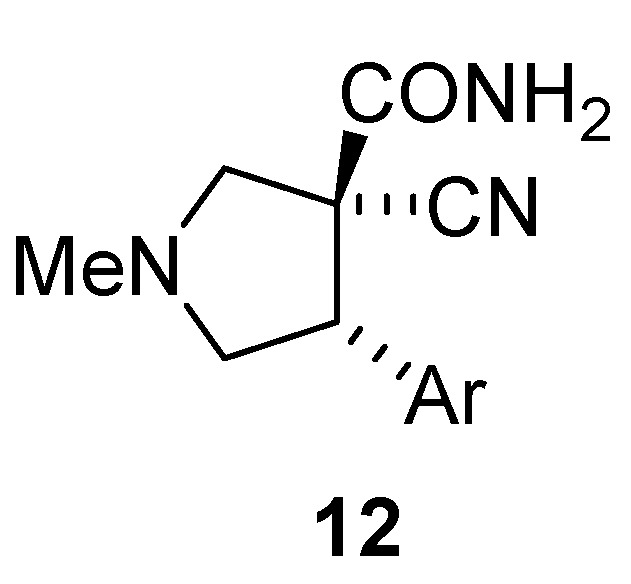
Amide 12 formed.

The chemoselective reduction of the nitrile was best achieved by a heterogeneous catalytic hydrogenation using Raney nickel under a hydrogen atmosphere at room temperature (Table 3) [44]. We found that the addition of NH_3_ or Et_3_N was crucial for the reaction to go to completion [45]. Eventually, Et_3_N was used since with NH_3_ amide **12** (Figure 3) was formed alongside the product.

Thus, compounds **7**{*1*–*6*} were reacted under these conditions to complete the synthesis of the library scaffolds. After elimination of Raney nickel by filtration through diatomaceous earth and evaporation of MeOH and Et_3_N, the reaction cleanly gave the α-aminomethyl esters **6**{*1*–*6*}.

**Table 3:** Reduction of the cyano group from the α-cyano esters **7**.

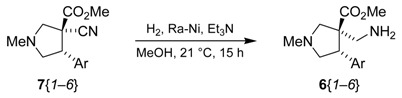
**Entry**	**Substrate**	**Product**	**Yield (%)**
1	**7**{*1*}	**6**{*1*}	95
2	**7**{*2*}	**6**{*2*}	95
3	**7**{*3*}	**6**{*3*}	89
4	**7**{*4*}	**6**{*4*}	85
5	**7**{*5*}	**6**{*5*}	73
6	**7**{*6*}	**6**{*6*}	95

### 2.4. Parallel synthesis of spiro dihydrouracils

**Figure 4:**
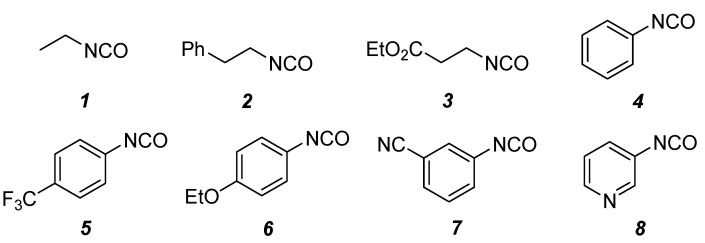
Isocyanates **13**{*1*–*8*} used for the reaction of scaffolds **6**{*1*–*6*}.

The procedure followed for the formation of the spiro dihydrouracils was formation of a urea by addition of an isocyanate and subsequent cyclization by reaction with a base. The conversion of chemset **6** into chemset **5** was accomplished using reagent chemset **13**. Eight isocyanates **13**{*1*–*8*} (alkyl, electron-rich aryl, electron-poor aryl, and heteroaryl isocyanates; Figure 4) were selected for the generation of a 48-compound library.

**Scheme 3:**
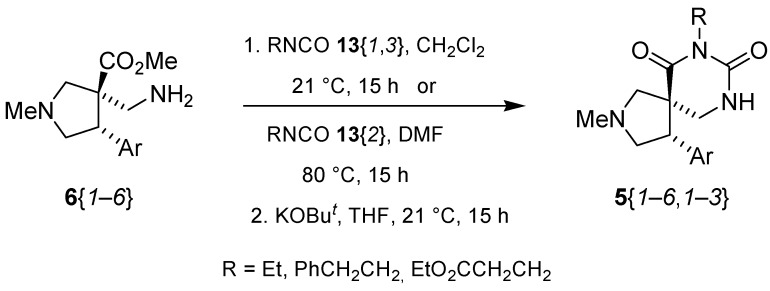
Spiro dihydrouracil formation from scaffolds **6**{*1*–*6*}.

**Scheme 4:**
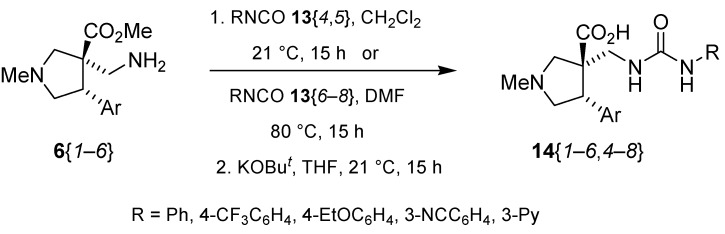
α-Ureidomethyl acid formation from scaffolds **6**{*1*–*6*}.

The reactions for the formation of the α-ureidomethyl esters were run in either CH_2_Cl_2_ or DMF depending on reagent solubility and reactivity. Thus, the reactions of **6**{*1*–*6*} and **13**{*1*,*3*–*5*} in CH_2_Cl_2_ for 15 h at room temperature afforded the corresponding α-ureidomethyl esters. In order to reach full conversion to the α-ureidomethyl esters using isocyanates **13**{*2*,*6*–*8*}, DMF at 80 °C for 15 h had to be used. After evaporation of the solvent, the crude mixture was dissolved in THF and 1 M KOBu*^t^* in THF (1 equiv) was added [46,47]. The reactions were stirred at room temperature for 15 h and the solvent was evaporated. Liquid–liquid extraction afforded two different types of compounds, depending on the isocyanate used: (1) the alkyl isocyanates **13**{*1*–*3*} gave the expected 4-aryl-spiro[dihydrouracil-5,3′-pyrrolidines] **5**{*1*–*6*,*1*–*3*} with yields ranging from 49 to 80% (61% average) and with purities ranging from 60 to 99% (83% average; Scheme 3 and Table 4) according to LC-MS analysis (also confirmed by ^1^H-NMR spectroscopy) and (2) the aryl isocyanates **13**{*4*–*8*} gave mostly the unexpected α-ureidomethyl acids **14**{*1*–*6*,*4*–*8*} with yields ranging from 45 to 79% (64% average) and with purities ranging from 0 to >99% (82% average; Scheme 4 and Table 5) according to LC-MS analysis (also confirmed by ^1^H-NMR spectroscopy).

**Figure 5:**
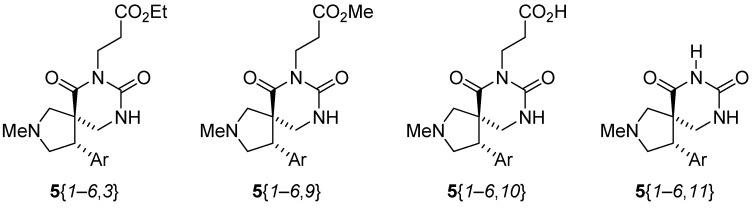
Products formed from the reaction with reagent **13**{*3*}.

The reactions carried out with reagent **13**{*1*} resulted in high purities (89% average) for the formation of the spiro dihydrouracils, due to lack of competing reactions. The purities of the products from the reactions run in DMF (reagent **13**{*2*}) were in the range 60 to 92% (70% average). These lower purities could be due to partial decomposition of the isocyanates at the temperature used for the reactions in DMF. The reactions carried out with reagent **13**{*3*} gave a mixture of the expected ethyl esters **5**{*1*–*6*,*3*}, the methyl esters **5**{*1*–*6*,*9*} (from transesterification of the ethyl ester on the R group by methoxide, formed in the cyclization), the acids **5**{*1*–*6*,*10*} (from hydrolysis of the esters), and the deorganylated compounds **5**{*1*–*6*,*11*} (Figure 5 and Table 4). The overall cyclization reaction worked well, since products **5**{*1*–*6*,*9*}, **5**{*1*–*6*,*10*}, and **5**{*1*–*6*,*11*} were formed from **5**{*1*–*6*,*3*}. Shorter reaction times should thus be used to avoid these side reactions.

**Scheme 5:**
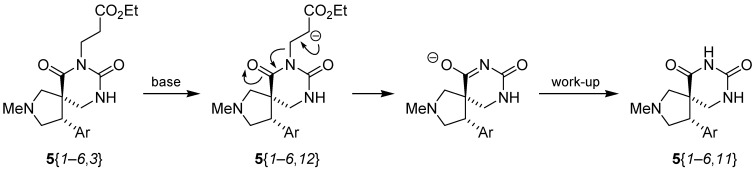
Formation of compounds **5**{*1*–*6*,*11*}.

The above-mentioned deorganylation side reaction could have taken place through an E1cB mechanism (Scheme 5). The substrate **5**{*1*–*6*,*3*} (or **5**{*1*–*6*,*9*}) is deprotonated to form the enolate **5**{*1*–*6*,*12*}, which undergoes an elimination reaction to afford **5**{*1*–*6*,*11*} after work-up.

The aryl-substituted dihydrouracils underwent hydrolysis (and not the alkyl-substituted dihydrouracils) because the electrophilicity of the ureide carbonyls is enhanced (with respect to the alkyl group) due to the conjugation of the imide-type nitrogen with the aryl group. Thus, residual H_2_O from KOBu*^t^* could have hydrolyzed the ureide to the ureido acid [48].

**Table 4:** Parallel synthesis of spiro dihydrouracil library **5**{*1*–*6*,*1*–*3*}^a,b^.

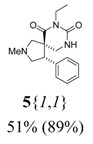	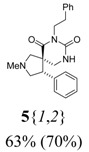	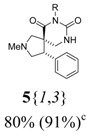
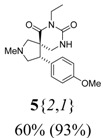	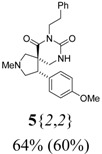	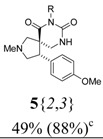
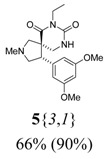	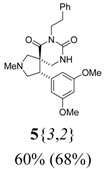	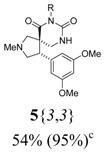
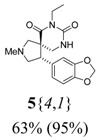	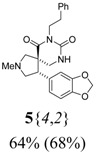	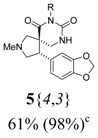
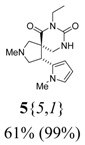	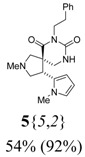	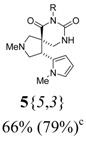
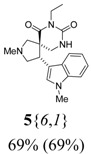	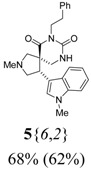	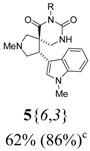

^a^ % = Crude yield based on mass recovery; ^b^ (%) = Purity determined by LC-MS at 215 nm; ^c^ Mixture of compounds, R = CH_2_CH_2_CO_2_Et, CH_2_CH_2_CO_2_Me, CH_2_CH_2_CO_2_H, and H.

**Table 5:** Parallel synthesis of α-ureidomethyl acid library **14**{*1*–*6*,*4*–*8*}^a,b^.

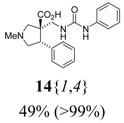	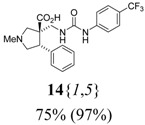	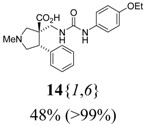	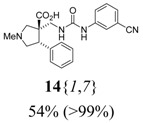	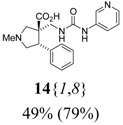
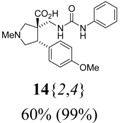	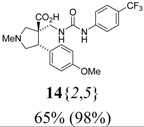	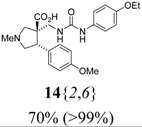	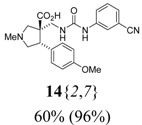	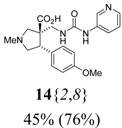
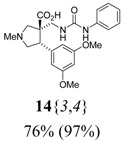	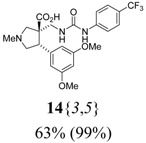	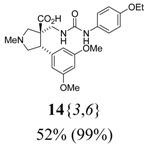	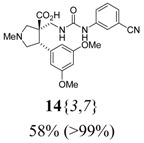	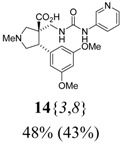
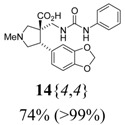	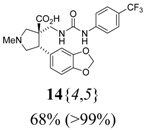	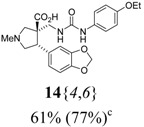	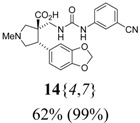	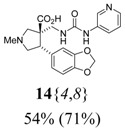
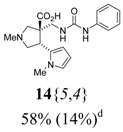	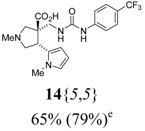	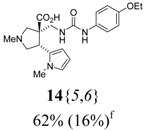	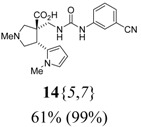	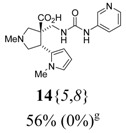
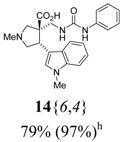	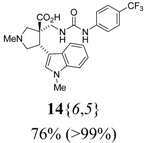	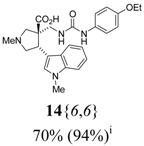	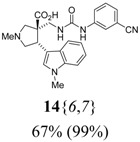	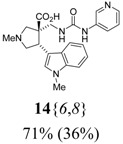

^a^ % = Crude yield based on mass recovery; ^b^ (%) = Purity determined by LC-MS at 215 nm; ^c^ Plus **5**{*4*,*6*} (21%). ^d^ Plus **5**{*5*,*4*} (77%); ^e^ Plus **5**{*5*,*5*} (18%). ^f^ Plus **5**{*5*,*6*} (81%); ^g^ Plus **5**{*5*,*8*} (54%); ^h^ Plus **5**{*6*,*4*} (2%); ^i^ Plus **5**{*6*,*6*} (5%).

In order to find conditions for the exclusive formation of the spiro dihydrouracils using aryl isocyanates, the α-ureidomethyl ester **15**{*1*,*4*} was synthesized, isolated, and reacted with several bases under different conditions for the formation of spiro dihydrouracil **5**{*1*,*4*} (Table 6).

Firstly, the reaction was attempted with an easy-to-handle base because, if successful, it would make the work-up of the reactions easy—an important factor in parallel synthesis. All the amines used were found to have insufficient basicity for this transformation to take place (entries 1–5) [49,50,51]. The amidine DBU gave promising results, but the separation of the product **5**{*1*,*4*} from DBU (and especially from the coreagent Bu_4_NBr, entry 8) was difficult and tedious, making these reaction conditions unsuitable for parallel synthesis [52,53]. Potassium *tert*-butoxide was the only base that caused >99% of the starting material to react [54], but it was the base that gave the largest amount of hydrolyzed product **14**{*1*,*4*} (entries 9–12). Heating only (entry 15) resulted in decomposition of the starting material. To the best of our knowledge, there is no example in the literature of a dihydrouracil ring with such a tendency toward hydrolysis under basic conditions. This cyclization can also take place using acid catalysis [55,56,57,58], but this has not yet been attempted.

**Table 6:** Optimization of the formation of spiro dihydrouracil **5**{*1*,*4*}.

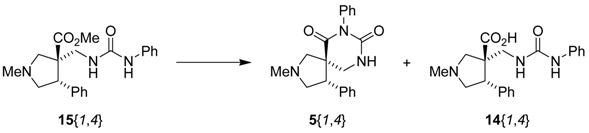
**Entry**	**Conditions**	**Products^a^ 15{*1*,*4*}/5{*1*,*4*}/14{*1*,*4*}**
1	Et_3_N (1.1 equiv), THF, Ar, reflux, 22 h	1:0:0
2	Proton sponge (0.2 equiv), THF, 21 °C, 5 h	1:0:0
3	Proton sponge (1 equiv), THF, 21 °C, 17 h	1:0:0
4	Proton sponge (1 equiv), THF, reflux, 7 h	1:0:0
5	DIPEA (1 equiv), DMF, 90 °C, 6 h	1:0:0
6	DBU (1 equiv), THF, Ar, 26 °C, 5 h	1:0:0
7	DBU (1 equiv), THF, Ar, reflux, 17 h	5:1:0
8	DBU (1 equiv), Bu_4_NBr, 4 Å MS, PhMe, Ar, reflux, 27 h	1:9:0
9	KOBu*^t^* (1 equiv), THF, 29 °C, 30 min	1:3:1
10	KOBu*^t^* (1 equiv), THF, 31 °C, 55 min	0:2:1
11	KOBu*^t^* (1 equiv), THF, Ar, 21 °C, 2 h	0:1:2
12	KOBu*^t^* (0.1 equiv), THF, Ar, 21 °C, 17 h	3:6:1
13	Phosphazene P_2_-*t*-Bu (0.1 equiv), THF, Ar, 21 °C, 5 h	1:3:0
14	Phosphazene P_2_-*t*-Bu (1 equiv), THF, Ar, 21 °C, 18 h	1:0:4
15	DMSO, 165 °C, 15 h	decomposition

^a^ Ratio calculated by integration of the ^1^H-NMR signals of the crude reaction mixture.

The compounds **5**{*1*–*6*,*1*–*3*}, **5**{*1*,*4*}, **6**{*1*–*6*}, **14**{*1*–*6*,*4*–*8*}, and **15**{*1*,*4*} were tested on different CNS targets, but the results cannot be published because of the patent policy of the companies involved in the project.

## 3. Experimental

### 3.1. General

Reagents were obtained from commercial suppliers and were used without purification. Solvents were distilled from appropriate drying agents prior to use and were stored under nitrogen. Reactions were followed, and *R*_F_ values were obtained, using thin-layer chromatography (TLC) on silica gel-coated plates (Merck 60 F254) with the indicated solvent mixture. Detection was performed with UV light and/or by charring at ca. 150 °C after dipping into a solution of KMnO_4_ or ninhydrin. Column or flash chromatography was carried out using ACROS silica gel (0.035–0.070 mm, pore diameter *ca.* 6 nm). IR spectra were recorded on an ATI Mattson Genesis Series FTIR spectrometer. High-resolution mass spectra were recorded on a JEOL AccuTOF (ESI) or a MAT900 (EI, CI, and ESI). Low-resolution ESI mass spectra were recorded on a Thermo Finnigan LCQ Advantage Max Ion Trap mass spectrometer. Elemental analyses were carried out using a Carlo Erba Instruments CHNS-O EA 1108 element analyzer. Melting points were analyzed with a Büchi melting point B-545 and are not corrected. Gas chromatography (GC) was performed on a Hewlett Packard 5890, containing a HP1 column (25 m x 0.32 mm x 0.17 μm), FID detection, and equipped with a HP3393A integrator. NMR spectra were recorded at 298 K on a Bruker DMX 300 (300 MHz) or a Varian 400 (400 MHz) spectrometer in the solvent indicated. Chemical shifts are given in parts per million (ppm) with respect to tetramethylsilane (0.00 ppm) or CD_3_SOCHD_2_ (2.50 ppm) as internal standard for ^1^H-NMR; and CDCl_3_ (77.16 ppm) or CD_3_SOCD_3_ (39.52 ppm) as internal standard for ^13^C-NMR [59]. Coupling constants are reported as *J* values in hertz (Hz). Multiplicity data are denoted by s (singlet), d (doublet), t (triplet), q (quartet), m (multiplet), b (broad), and app (apparent). Peak assignment in ^13^C spectra are based on 2D gHSQC and gHMBC spectra, and DEPT 135 when needed. Chain numbering corresponds to IUPAC nomenclature, so unprimed atoms belong to the principal chain, primed atoms belong to the first named substituent, doubled-primed atoms to the second named substituent, etc. LC-MS measurements were run on a Shimadzu LC-10A VP series liquid chromatography system, equipped with an SPD-10A VP UV-vis detector and a LCMS-2010A mass spectrometer. The column used for the LC analysis was an Agilent Zorbax Extend C18 (3.5 μm, 4.6 × 150 mm), and it was eluted at 1 mL/min with a gradient made up of two solvent mixtures. Solvent A consisted of 0.1% trifluoroacetic acid in water and solvent B consisted of 0.1% trifluoroacetic acid in acetonitrile. The gradient was run as follows: *t* ) 0 min, 50% A; *t* ) 5 min, 5% A; *t* ) 10 min, 5% A; *t* ) 12.5 min, 50% A; *t* ) 20 min, 50% A. A wavelength of 215 nm was selected for the analysis of purity.

### 3.2. General procedure for Knoevenagel condensation reaction

Piperidine (5 drops) was added to a solution of methyl 2-cyanoacetate (**9**) and the aldehyde **10** (1.0 equiv) in MeOH. The resulting reaction mixture was stirred at room temperature for the time indicated in each case. The reaction mixture was filtered and the precipitate was recrystalized from MeOH. The filtrate was concentrated under reduced pressure and purified by recrystalization from MeOH.

*Methyl (E)-2-cyano-3-phenylacrylate* (**8**{*1*}): According to the general procedure, the reaction of methyl 2-cyanoacetate (**9**, 7.940 g, 80.13 mmol) with benzaldehyde **10**{*1*} (8.504 g, 80.13 mmol) over 30 min afforded **8**{*1*} (14.910 g, 99%) as a white solid. ^1^H-NMR [400 MHz, δ (ppm), CDCl3]: 8.21 (s, 1 ^1^H, 3-C*H*), 7.97–7.92 (m, 2 ^1^H, 2′-C*H* + 6′-C*H*), 7.56–7.43 (m, 3 ^1^H, 3′-C*H* + 4′-C*H* + 5′-C*H*), 3.90 (s, 3 ^1^H, OC*H*_3_). ^13^C-NMR [75 MHz, δ (ppm), CDCl3]: 162.5 (*C*O_2_), 154.9 (3-*C*), 133.1 (4′-*C*), 131.1 (1′-*C*), 130.8 (2′-*C* + 6′-*C*), 129.0 (3′-*C* + 5′-*C*), 115.2 (*C*N), 102.4 (2-*C*), 53.4 (O*C*H_3_). FTIR [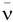 (cm^–1^), neat]: 3036, 2954, 2224, 1727, 1606, 1200, 767, 685. Elem. anal. calcd. for C_11_H_9_NO_2_: C 70.58%, H 4.85%, N 7.48%; found C 70.39%, H 4.54%, N 7.43%. *R*_F_: 0.63 (heptane/AcOEt, 1:1). Mp: 87.9 °C (from MeOH, colorless flake-like crystals). Purity: >99.5% (GC).

*Methyl (E)-2-cyano-3-(4-methoxyphenyl)acrylate* (**8**{*2*}): According to the general procedure, the reaction of methyl 2-cyanoacetate (**9**, 6.804 g, 68.66 mmol) with 4-methoxybenzaldehyde **10**{*2*} (9.349 g, 68.66 mmol) over 30 min afforded **8**{*2*} (14.020 g, 94%) as a white solid. ^1^H-NMR [400 MHz, δ (ppm), CDCl3]: 8.14 (s, 1 ^1^H, 3-C*H*), 8.00–7.95 (m, 2 ^1^H, 2′-C*H* + 6′-C*H*), 7.01–6.95 (m, 2 ^1^H, 3′-C*H* + 5′-C*H*), 3.91 (s, 3 ^1^H, CO_2_C*H*_3_), 3.88 (s, 3 ^1^H, OC*H*_3_). ^13^C-NMR [75 MHz, δ (ppm), CDCl3]: 163.8 (4′-*C*), 163.5 (*C*O_2_), 154.5 (3-*C*), 133.6 (2′-*C* + 6′-*C*), 124.2 (1′-*C*), 116.1 (*C*N), 114.7 (3′-*C* + 5′-*C*), 98.8 (2-*C*), 55.6 (O*C*H_3_), 53.1 (CO_2_*C*H_3_). FTIR [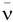 (cm^–1^), neat]: 3084, 2954, 2846, 2215, 1714, 1580, 1264, 1174, 841. Elem. anal. calcd. for C_12_H_11_NO_3_: C 66.35%, H 5.10%, N 6.45%; found C 66.24%, H 4.98%, N 6.33%. *R*_F_: 0.58 (heptane/AcOEt, 1:1). Mp: 104.1 °C (from MeOH, off-white small crystals). Purity: >99.5% (GC).

*Methyl (E)-2-cyano-3-(3,5-dimethoxyphenyl)acrylate* (**8**{*3*}): According to the general procedure, the reaction of methyl 2-cyanoacetate (**9**, 5.835 g, 58.89 mmol) with 3,5-dimethoxybenzaldehyde **10**{*3*} (9.785 g, 58.89 mmol) over 40 min afforded **8**{*3*} (12.399 g, 99%) as a yellow solid. ^1^H-NMR [400 MHz, δ (ppm), CDCl3]: 8.17 (s, 1 ^1^H, 3-C*H*), 7.15 (d, *J* = 2.4 Hz, 2 ^1^H, 2′-C*H* + 6′-C*H*), 6.65 (t, *J* = 2.4 Hz, 1 ^1^H, 4′-C*H*), 3.94 (s, 3 ^1^H, CO_2_C*H*_3_), 3.84 (s, 6 ^1^H, 2 × OC*H*_3_). ^13^C-NMR [75 MHz, δ (ppm), CDCl3]: 163.1 (*C*O_2_), 161.2 (3′-*C* + 5′-*C*), 155.6 (3-*C*), 133.1 (1′-*C*), 115.6 (*C*N), 108.7 (2′-*C* + 6′-*C*), 106.4 (4′-*C*), 103.0 (2-*C*), 55.8 (2 × O*C*H_3_), 53.6 (CO_2_*C*H_3_). FTIR [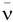 (cm^–1^), neat]: 3086, 2940, 2841, 2217, 1723, 1604, 1247, 1167, 840. Elem. anal. calcd for C_13_H_13_NO_4_: C 63.15%, H 5.30%, N 5.66%; found C 63.22%, H 5.14%, N 5.53%. *R*_F_: 0.56 (heptane/AcOEt, 1:1). Mp: 121.5 °C (from MeOH, long light yellow needles). Purity: >99.5% (GC).

*Methyl (E)-3-(1,3-benzodioxol-5-yl)-2-cyanoacrylate* (**8**{*4*}): According to the general procedure, the reaction of methyl 2-cyanoacetate (**9**, 6.002 g, 60.57 mmol) with piperonal **10**{*4*} (9.094 g, 60.57 mmol) over 60 min afforded **8**{*4*} (13.836 g, 99%) as a light greenish solid. ^1^H-NMR [400 MHz, δ (ppm), CDCl3]: 8.12 (s, 1 ^1^H, 3-C*H*), 7.71 (d, *J* = 1.9 Hz, 1 ^1^H, 4′-C*H*), 7.41 (ddd; *J* = 8.2, 1.9, 0.6 Hz; 1 ^1^H, 6′-C*H*), 6.91 (d, *J* = 8.2 Hz, 1 ^1^H, 7′-C*H*), 6.09 (s, 2 ^1^H, 2′-C*H*_2_), 3.92 (s, 3 ^1^H, OC*H*_3_). ^13^C-NMR [75 MHz, δ (ppm), CDCl3]: 163.6 (*C*O_2_), 154.7 (3-*C*), 152.5 (7′a-*C*), 148.8 (3′a-*C*), 130.0 (6′-*C*), 126.1 (5′-*C*), 116.1 (*C*N), 109.1 (4′-*C*), 109.0 (7′-*C*), 102.5 (2′-*C*), 99.5 (2-*C*), 53.4 (O*C*H_3_). FTIR [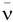 (cm^–1^), neat]: 3029, 2957, 2218, 1723, 1579, 1244, 1205, 1041, 922, 819. HRMS [EI (m/z)] calcd for C_12_H_9_NO_4_ = 231.0532, found for [M^+•^] = 231.0532 (|Δ| = 0.0 ppm), peaks at (relative intensity): 231 (100), 200 (21), 170 (32), 142 (12), 114 (21). Elem. anal. calcd for C_12_H_9_NO_4_: C 62.34%, H 3.92%, N 6.06%; found C 62.24%, H 3.86%, N 6.00%. *R*_F_: 0.60 (heptane/AcOEt, 1:1). Mp: 169.6 °C (from MeOH, light green cotton-like solid). Purity: >99.5% (GC).

*Methyl (E)-2-cyano-3-(1-methyl-1H-pyrrol-2-yl)acrylate* (**8**{*5*}): According to the general procedure, the reaction of methyl 2-cyanoacetate (**9**, 7.107 g, 71.72 mmol) with 1-methyl-1*H*-pyrrole-2-carbaldehyde **10**{*5*} (7.827 g, 71.72 mmol) over 120 min afforded **8**{*5*} (12.932 g, 95%) as a yellow solid. ^1^H-NMR [400 MHz, δ (ppm), CDCl3]: 8.08 (s, 1 ^1^H, 3-C*H*). 7.72 (d, *J* = 4.4 Hz, 1 ^1^H, 3′-C*H*), 7.01 (t, *J* = 1.8 Hz, 1 ^1^H, 5′-C*H*), 6.39–6.36 (m, 1 ^1^H, 4′-C*H*), 3.89 (s, 3 ^1^H, OC*H*_3_), 3.78 (s, 3 ^1^H, NC*H*_3_). ^13^C-NMR [75 MHz, δ (ppm), CDCl3]: 164.8 (*C*O_2_), 139.6 (3-*C*), 131.7 (5′-*C*), 127.5 (2′-*C*), 120.0 (3′-*C*), 117.1(*C*N), 112.2 (4′-*C*), 92.8 (2-*C*), 53.0 (O*C*H_3_), 34.4 (N*C*H_3_). FTIR [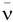 (cm^–1^), neat]: 3120, 2957, 2899, 2210, 1717, 1591, 1243, 748. Elem. anal. calcd for C_10_H_10_N_2_O_2_: C 63.15%, H 5.30%, N 14.73%; found C 63.19%, H 5.09%, N 14.70%. *R*_F_: 0.29 (heptane/AcOEt, 1:1). Mp: 154.2 °C (from MeOH, yellow solid). Purity: >99.5% (GC).

*Methyl (E)-2-cyano-3-(1-methyl-1H-indol-3-yl)acrylate* (**8**{*6*}): According to the general procedure, the reaction of methyl 2-cyanoacetate (**9**, 6.011 g, 60.66 mmol) with 1-methyl-1*H*-indole-3-carbaldehyde **10**{*6*} (9.656 mg, 60.66 mmol) over 120 min afforded **8**{*6*} (13.977 g, 96%) as a yellow solid. ^1^H- NMR [400 MHz, δ (ppm), CDCl3]: 8.56 (s, 1 ^1^H, 3-C*H*), 8.49 (s, 1 ^1^H, 2′-C*H*), 7.84–7.80 (m, 1 ^1^H, 4′-C*H*), 7.42–7.31 (m, 3 ^1^H, 5′-C*H* + 6′-C*H* + 7′-C*H*), 3.91 (s, 3 ^1^H, CO_2_C*H*_3_), 3.90 (s, 3 ^1^H, NC*H*_3_). ^13^C- NMR [75 MHz, δ (ppm), CDCl3]: 164.6 (*C*O_2_), 146.2 (3-*C*), 137.0 (7′a-*C*), 134.9 (2′-*C*), 128.5 (3′a-*C*), 124.1 (6′-*C*), 122.8 (5′-*C*), 118.6 (4′-*C*), 118.5 (*C*N), 110.6 (7′-*C*), 110.1 (3′-*C*), 93.3 (2-*C*), 52.9 (O*C*H_3_), 34.2 (N*C*H_3_). FTIR [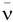 (cm^–1^), neat]: 3118, 3026, 2950, 2222, 1701, 1586, 1255, 751. HRMS [EI (m/z)] calcd for C_14_H_12_N_2_O_2_ = 240.0899, found for [M^+•^] = 240.0895 (|Δ| = 1.6 ppm), peaks at (relative intensity): 240 (100), 209 (53), 140 (18), 49 (12). Elem. anal. calcd for C_14_H_12_N_2_O_2_: C 69.99%, H 5.03%, N 11.66%; found C 69.69%, H 4.85%, N 11.42%. *R*_F_: 0.37 (heptane/AcOEt, 1:1). Mp: 165.9 °C (from MeOH, yellow solid). Purity: >99.5% (GC).

*Methyl (E)-2-cyano-3-(1H-pyrrol-2-yl)acrylate* (**8**{*7*}): According to the general procedure, the reaction of methyl 2-cyanoacetate (**9**, 991 mg, 10.00 mmol) with 1*H*-pyrrole-2-carbaldehyde **10**{*7*} (951 mg, 10.00 mmol) over 30 min afforded **8**{*7*} (1.744 g, 99%) as a yellow solid. ^1^H-NMR [400 MHz, δ (ppm), CDCl3]: 9.92 (bs, 1 ^1^H, N*H*), 8.02 (s, 1 ^1^H, 3-C*H*), 7.26–7.23 (m, 1 ^1^H, 5′-C*H*), 6.98–6.94 (m, 1 ^1^H, 3′-C*H*), 6.44 (dt; *J* = 3.9, 2.3 Hz; 1 ^1^H, 4′-C*H*), 3.89 (s, 3 ^1^H, OC*H*_3_). ^13^C-NMR [75 MHz, δ (ppm), CDCl3]: 164.2 (*C*O_2_), 142.8 (3-*C*), 128.4 (5′-*C*), 127.0 (2′-*C*), 124.7 (3′-*C*), 118.6 (*C*N), 112.7 (4′-*C*), 91.8 (2-*C*), 53.0 (O*C*H_3_). FTIR [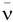 (cm^–1^), neat]: 3300, 3032, 2950, 2215, 1692, 1593, 1221, 750. Elem. anal. calcd for C_9_H_8_N_2_O_2_: C 61.36%, H 4.58%, N 15.90%; found C 61.45%, H 4.58%, N 15.79%. *R*_F_: 0.37 (heptane/AcOEt, 1:1). Mp: 140.2 °C (from MeOH, small thin yellow needles). Purity: >99.5% (GC).

*Methyl (E)-2-cyano-3-(1H-indol-3-yl)acrylate* (**8**{*8*}): According to the general procedure, the reaction of methyl 2-cyanoacetate (**9**, 991 mg, 10.00 mmol) with 1*H*-indole-3-carbaldehyde **10**{*8*} (1.452 g, 10.00 mmol) in THF (15 mL) over 480 min afforded **8**{*8*} (2.239 g, 99%) as a yellow solid. ^1^H-NMR [400 MHz, δ (ppm), CD3SOCD3]: 12.64 (bs, 1 ^1^H, N*H*), 8.64 (s, 1 ^1^H, 2′-C*H*), 8.63 (s, 1 ^1^H, 3-C*H*), 8.05–7.99 (m, 1 ^1^H, 4′-C*H*), 7.67–7.63 (m, 1 ^1^H, 7′-C*H*), 7.41–7.30 (m, 2 ^1^H, 5′-C*H* + 6′-C*H*), 3.92 (s, 3 ^1^H, OC*H*_3_). ^13^C-NMR [75 MHz, δ (ppm), CD3SOCD3]: 163.3 (*C*O_2_), 146.3 (3-*C*), 135.9 (7′a-*C*), 132.4 (2′-*C*), 126.6 (3′a-*C*), 123.3 (6′-*C*), 121.9 (5′-*C*), 118.3 (4′-*C*), 117.8 (*C*N), 112.7 (7′-*C*), 109.7 (3′-*C*), 91.8 (2-*C*), 52.6 (O*C*H_3_). FTIR [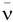 (cm^–1^), neat]: 3266, 3132, 2989, 2943, 2212, 1695, 1589, 1241, 742. Elem. anal. calcd for C_13_H_10_N_2_O_2_: C 69.02%, H 4.46%, N 12.38%; found C 68.97%, H 4.38%, N 12.22%. *R*_F_: 0.80 (AcOEt). Mp: 189.5 °C (from MeOH, yellow needles). Purity: >99.5% (GC).

*Methyl (E)-2-cyano-3-(2-thienyl)acrylate* (**8**{*9*}): According to the general procedure, the reaction of methyl 2-cyanoacetate (**9**, 991 mg, 10.00 mmol) with thiophene-2-carbaldehyde **10**{*9*} (1.121 g, 10.00 mmol) over 30 min afforded **8**{*9*} (1.816 g, 94%) as a light brown solid. ^1^H-NMR [400 MHz, δ (ppm), CDCl3]: 8.36 (s, 1 ^1^H, 3-C*H*), 7.84 (d, *J* = 3.9 Hz, 1 ^1^H, 3′-C*H*), 7.80 (d, *J* = 4.9 Hz, 1 ^1^H, 5′-C*H*), 7.24 (dd; *J* = 4.9, 3.9 Hz; 1 ^1^H, 4′-C*H*), 3.92 (s, 3 ^1^H, OC*H*_3_). ^13^C-NMR [75 MHz, δ (ppm), CDCl3]: 162.9 (*C*O_2_), 146.7 (3-*C*), 137.2 (3′-*C*), 135.8 (2′-*C*), 135.2 (5′-*C*), 128.6 (4′-*C*), 115.6 (*C*N), 98.8 (2-*C*), 53.4 (O*C*H_3_). FTIR [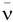 (cm^–1^), neat]: 3087, 3028, 2964, 2216, 1718, 1590, 1271, 1214, 729. Elem. anal. calcd for C_9_H_7_NO_2_S: C 55.94%, H 3.65%, N 7.25%; found C 55.98%, H 3.63%, N 7.23%. *R*_F_: 0.61 (heptane/AcOEt, 1:1). Mp: 106.9 °C (from MeOH, light brown needles). Purity: >99.5% (GC).

*Methyl (E)-2-cyano-3-(2-pyridyl)acrylate* (**8**{*10*}): According to the general procedure, the reaction of methyl 2-cyanoacetate (**9**, 991 mg, 10.00 mmol) with pyridine-2-carbaldehyde **10**{*10*} (1.071 g, 10.00 mmol) over 30 min afforded **8**{*10*} (1.863 g, 99%) as a brown solid. ^1^H-NMR [400 MHz, δ (ppm), CDCl3]: 8.83 (ddd; *J* = 4.8, 1.6, 1.0 Hz; 1 ^1^H, 6′-C*H*), 8.30 (s, 1 ^1^H, 3-C*H*), 7.89 (app dt; *J* = 7.8, 1.2 Hz; 1 ^1^H, 3′-C*H*), 7.85 (app dt; *J* = 1.6, 7.8 Hz; 1 ^1^H, 4′-C*H*), 7.44 (ddd; *J* = 7.3, 4.9, 1.5 Hz; 1 ^1^H, 5′-C*H*), 3.96 (s, 3 ^1^H, OC*H*_3_). ^13^C-NMR [75 MHz, δ (ppm), CDCl3]: 162.6 (*C*O_2_), 153.7 (3-*C*), 150.7 (6′-*C*), 150.0 (2′-*C*), 137.1 (4′-*C*), 126.8 (5′-*C*), 126.4 (3′-*C*), 114.8 (*C*N), 106.4 (2-*C*), 53.7 (O*C*H_3_). FTIR [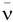 (cm^–1^), neat]: 3050, 2963, 2220, 1720, 1278, 1215, 781, 740. Elem. anal. calcd for C_10_H_8_N_2_O_2_: C 63.83%, H 4.28%, N 14.89%; found C 64.06%, H 4.26%, N 14.79%. *R*_F_: 0.34 (heptane/AcOEt, 1:1). Mp: 131.7 °C (from MeOH, small brown needles). Purity: >99.5% (GC).

*Methyl (E)-2-cyano-3-(3-pyridyl)acrylate* (**8**{*11*}): According to the general procedure, the reaction of methyl 2-cyanoacetate (**9**, 991 mg, 10.00 mmol) with pyridine-3-carbaldehyde **10**{*11*} (1.071 g, 10.00 mmol) over 90 min afforded **8**{*11*} (1.750 g, 93%) as a light yellow dust. ^1^H-NMR [300 MHz, δ (ppm), CDCl3]: 8.91 (d, *J* = 1.8 Hz, 1 ^1^H, 2′-C*H*), 8.74 (dd; *J* = 4.8, 1.8 Hz; 1 ^1^H, 6′-C*H*), 8.57–8.51 (m, 1 ^1^H, 4′-C*H*), 8.26 (s, 1 ^1^H, 3-C*H*), 7.46 (dd; *J* = 7.8, 4.8 Hz; 1 ^1^H, 5′-C*H*), 3.95 (s, 3 ^1^H, OC*H*_3_). ^13^C-NMR [75 MHz, δ (ppm), CDCl3]: 161.9 (*C*O_2_), 153.3 (3-*C*), 152.7 (6′-*C*), 151.3 (2′-*C*), 135.8 (4′-*C*), 127.3 (3′-*C*), 123.9 (5′-*C*), 114.7 (*C*N), 105.1 (2-*C*), 53.8 (O*C*H_3_). FTIR [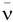 (cm^–1^), neat]: 3031, 2959, 2223, 1722, 1280, 697. *R*_F_: 0.13 (heptane/AcOEt, 1:1). Mp: 129.2 °C.

*Methyl (E)-2-cyano-3-(4-pyridyl)acrylate* (**8**{*12*}): According to the general procedure, the reaction of methyl 2-cyanoacetate (**9**, 991 mg, 10.00 mmol) with pyridine-4-carbaldehyde **10**{*12*} (1.071 g, 10.00 mmol) over 25 min afforded **8**{*12*} (1.863 g, 99%) as a pink solid. ^1^H-NMR [400 MHz, δ (ppm), CDCl3]: 8.86–8.80 (m, 2 ^1^H, 2′-C*H* + 6′-C*H*), 8.23 (s, 1 ^1^H, 3-C*H*), 7.80–7.74 (m, 2 ^1^H, 3′-C*H* + 5′-C*H*), 3.97 (s, 3 ^1^H, OC*H*_3_). ^13^C-NMR [75 MHz, δ (ppm), CDCl3]: 161.8 (*C*O_2_), 152.4 (3-*C*), 151.2 (2′-*C* + 6′-*C*), 138.0 (4′-*C*), 123.3 (3′-*C* + 5′-*C*), 114.2 (*C*N), 107.8 (2-*C*), 53.8 (O*C*H_3_). FTIR [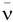 (cm^–1^), neat]: 3033, 2955, 2225, 1726, 1236, 1198, 818. Elem. anal. calcd for C_10_H_8_N_2_O_2_: C 63.83%, H 4.28%, N 14.89%; found C 63.94%, H 4.25%, N 14.75%. *R*_F_: 0.12 (heptane/AcOEt, 1:1). Mp: 126.6 °C (from MeOH, small pink needles). Purity: >99.5% (GC).

### 3.3. General procedure for 1,3-dipolar cycloaddition reactions of ***8***

A round-bottomed flask fitted with a Dean–Stark apparatus, a reflux condenser, and a drying tube containing calcium chloride was charged with 2-cyanoacrylate **8** and toluene (0.20–0.25 M). When the mixture was under reflux, sarcosine (*N*-methylglycine; 1.2 equiv) and paraformaldehyde (3.6 equiv) were added. This addition was repeated every 40 min until the substrate had completely reacted. Water (20 mL) was then added and the layers were separated. The aqueous layer was extracted with Et_2_O (3 × 30 mL) and the combined organic layers were dried (MgSO_4_), filtered, and concentrated *in vacuo*.

*(±)-Methyl (3R,4R)-3-cyano-1-methyl-4-phenylpyrrolidine-3-carboxylate* (**7**{*1*}): According to the general procedure, 2-cyanoacrylate **8**{*1*} (5.342 g, 28.54 mmol) afforded **7**{*1*} (6.524 g, 94%) as a white solid, after column chromatography (heptane/AcOEt, 3:1°2:1). ^1^H-NMR [400 MHz, δ (ppm), CDCl3]: 7.33–7.19 (m, 5 ^1^H, Ph), 4.00 (app t, *J* = 7.8 Hz, 1 ^1^H, 4-C*H*), 3.70 (s, 3 ^1^H, OC*H*_3_), 3.25 (d, *J* = 9.9 Hz, 1 ^1^H, 2-CH*H*), 3.18 (d, *J* = 9.9 Hz, 1 ^1^H, 2-C*H*H), 3.04 (dd; *J* = 9.6, 7.8 Hz; 1 ^1^H, 5-CH*H*), 3.00 (dd; *J* = 9.6, 8.1 Hz; 1 ^1^H, 5-C*H*H), 2.38 (s, 3 ^1^H, NC*H*_3_). ^13^C-NMR [75 MHz, δ (ppm), CDCl3]: 167.6 (*C*O_2_), 136.6 (1′-*C*), 128.0 (2′-*C* + 6′-*C*), 127.9 (3′-*C* + 5′-*C*), 127.5 (4′-*C*), 117.2 (*C*N), 64.9 (2-*C*), 60.2 (5-*C*), 54.4 (3-*C*), 53.5 (CO_2_*C*H_3_), 51.8 (4-*C*), 41.1 (N*C*H_3_). FTIR [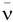 (cm^–1^), neat]: 2950, 2846, 2790, 2241, 1740, 1247, 772, 699. MS [ESI (m/z)] calcd for (C_14_H_16_N_2_O_2_ + H)^+^ = 245, found 245. *R*_F_: 0.24 (heptane/AcOEt, 1:1). Mp: 53.3 °C (from heptane, colorless crystals). Purity: >99.5% (GC).

*(±)-Methyl (3R,4R)-3-cyano-4-(4-methoxyphenyl)-1-methylpyrrolidine-3-carboxylate* (**7**{*2*}): According to the general procedure, 2-cyanoacrylate **8**{*2*} (6.162 g, 28.37 mmol) afforded **7**{*2*} (7.688 g, 99%) as a yellow oil. ^1^H-NMR [400 MHz, δ (ppm), CDCl3]: 7.28–7.22 (m, 2 ^1^H, 2′-C*H* + 6′-C*H*), 6.87–6.82 (m, 2 ^1^H, 3′-C*H* + 5′-C*H*), 3.98 (app t, *J* = 8.0 Hz, 1 ^1^H, 4-C*H*), 3.80 (s, 3 ^1^H, CO_2_C*H*_3_), 3.76 (s, 3 ^1^H, OC*H*_3_), 3.31 (d, *J* = 9.9 Hz, 1 ^1^H, 2-CH*H*), 3.20 (d, *J* = 9.9 Hz, 1 ^1^H, 2-C*H*H), 3.07 (dd; *J* = 9.6, 7.8 Hz; 1 ^1^H, 5-CH*H*), 3.02 (dd; *J* = 9.6, 8.1 Hz; 1 ^1^H, 5-C*H*H), 2.44 (s, 3 ^1^H, NC*H*_3_). ^13^C-NMR [75 MHz, δ (ppm), CDCl3]: 168.1 (*C*O_2_), 159.1 (4′-*C*), 129.4 (2′-*C* + 6′-*C*), 128.6 (1′-*C*), 117.8 (*C*N), 113.8 (3′-*C* + 5′-*C*), 65.2 (2-*C*), 60.8 (5-*C*), 55.2 (O*C*H_3_), 54.9 (3-*C*), 53.9 (CO_2_*C*H_3_), 51.8 (4-*C*), 41.6 (N*C*H_3_). FTIR [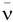 (cm^–1^), neat]: 2951, 2836, 2788, 2243, 1740, 1247, 832. MS [ESI (m/z)] calcd for (C_15_H_18_N_2_O_3_ + H)^+^ = 275, found 275. *R*_F_: 0.23 (heptane/AcOEt, 1:1). Purity: 98.9% (GC).

*(±)-Methyl (3R,4R)-3-cyano-4-(3,5-dimethoxyphenyl)-1-methylpyrrolidine-3-carboxylate* (**7**{*3*}): According to the general procedure, 2-cyanoacrylate **8**{*3*} (9.745 g, 39.41 mmol) afforded **7**{*3*} (11.398 g, 95%) as a white solid, after column chromatography (heptane/AcOEt, 3:1°2:1). ^1^H-NMR [400 MHz, δ (ppm), CDCl3]: 6.51 (d, *J* = 2.2 Hz, 2 ^1^H, 2′-C*H* + 6′-C*H*), 6.41 (t, *J* = 2.2 Hz, 1 ^1^H, 4′-C*H*), 3.99 (app t, *J* = 7.9 Hz, 1 ^1^H, 4-C*H*), 3.84 (s, 3 ^1^H, CO_2_C*H*_3_), 3.78 (s, 6 ^1^H, 2 × OC*H*_3_), 3.30 (d, *J* = 9.9 Hz, 1 ^1^H, 2-CH*H*), 3.24 (d, *J* = 9.9 Hz, 1 ^1^H, 2-C*H*H), 3.09 (dd; *J* = 9.6, 7.8 Hz; 1 ^1^H, 5-CH*H*), 3.06 (dd; *J* = 9.6, 8.0 Hz; 1 ^1^H, 5-C*H*H), 2.47 (s, 3 ^1^H, NC*H*_3_). ^13^C-NMR [75 MHz, δ (ppm), CDCl3]: 168.8 (*C*O_2_), 161.1 (3′-*C* + 5′-*C*), 139.4 (1′-*C*), 118.1 (*C*N), 107.0 (2′-*C* + 6′-*C*), 100.2 (4′-*C*), 65.7 (2-*C*), 60.7 (5-*C*), 55.6 (2 × O*C*H_3_), 54.8 (3-*C*), 54.2 (CO_2_*C*H_3_), 52.6 (4-*C*), 41.8 (N*C*H_3_). FTIR [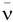 (cm^–1^), neat]: 2948, 2837, 2790, 2243, 1743, 1595, 1203, 1155. HRMS [EI (m/z)] calcd for C_16_H_20_N_2_O_4_ = 304.1423, found for [M^+•^] = 304.1436 (|Δ| = 4.2 ppm), peaks at (relative intensity): 304 (16), 261 (18), 193 (11), 57 (100), 42 (20). Elem. anal. calcd for C_16_H_20_N_2_O_4_: C 63.14%, H 6.62%, N 9.20%; found C 63.33%, H 6.65%, N 9.19%. *R*_F_: 0.19 (heptane/AcOEt, 1:1). Mp: 94.7 °C (from heptane, colorless crystals). Purity: >99.5% (GC).

*(±)-Methyl (3R,4R)-4 -(1,3-benzodioxol-5-yl)-3-cyano-1-methylpyrrolidine-3-carboxylate* (**7**{*4*}): According to the general procedure, 2-cyanoacrylate **8**{*4*} (9.774 g, 42.27 mmol) afforded **7**{*4*} (12.041 g, 99%) as a white solid. ^1^H-NMR [400 MHz, δ (ppm), CDCl3]: 6.88 (d, *J* = 1.7 Hz, 1 ^1^H, 4′-C*H*), 6.81 (dd; *J* = 8.0, 1.7 Hz; 1 ^1^H, 6′-C*H*), 6.77 (d, *J* = 8.0 Hz, 1 ^1^H, 7′-C*H*), 5.95 (d, *J* = 1.6 Hz, 1 ^1^H, 2′-CH*H*), 5.94 (d, *J* = 1.6 Hz, 1 ^1^H, 2′-C*H*H), 3.97 (t, *J* = 7.8 Hz, 1 ^1^H, 4-C*H*), 3.84 (s, 3 ^1^H, OC*H*_3_), 3.28 (d, *J* = 9.9 Hz, 1 ^1^H, 2-CH*H*), 3.23 (d, *J* = 9.9 Hz, 1 ^1^H, 2-C*H*H), 3.06 (dd; *J* = 9.5, 7.8 Hz; 1 ^1^H, 5-CH*H*), 3.01 (dd; *J* = 9.5, 7.8 Hz; 1 ^1^H, 5-C*H*H), 2.45 (s, 3 ^1^H, NC*H*_3_). ^13^C-NMR [75 MHz, δ (ppm), CDCl3]: 168.5 (*C*O_2_), 147.9 (3′a-*C*), 147.5 (7′a-*C*), 130.8 (5′-*C*), 122.0 (6′-*C*), 117.9 (*C*N), 108.8 (7′-*C*), 108.3 (4′-*C*), 101.2 (2′-*C*), 65.3 (2-*C*), 60.8 (5-*C*), 54.8 (3-*C*), 54.0 (O*C*H_3_), 52.1 (4-*C*), 41.5 (N*C*H_3_). FTIR [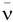 (cm^–1^), neat]: 2965, 2900, 2788, 2242, 1740, 1249, 1036, 929. HRMS [EI (m/z)] calcd for C_15_H_16_N_2_O_4_ = 288.1110, found for [M^+•^] = 288.1097 (|Δ| = 4.5 ppm), peaks at (relative intensity): 288 (13), 57 (100), 42 (11). Elem. anal. calcd for C_15_H_16_N_2_O_4_: C 62.49%, H 5.59%, N 9.72%; found C 62.56%, H 5.61%, N 9.69%. *R*_F_: 0.22 (heptane/AcOEt, 1:1). Mp: 87.6 °C (from heptane, colorless crystals). Purity: >99.5% (GC).

*(±)-Methyl (3R,4R)-3-cyano-1-methyl-4-(1-methyl-1H-pyrrol-2-yl)pyrrolidine-3-carboxylate* (**7**{*5*}): According to the general procedure, 2-cyanoacrylate **8**{*5*} (1.132 g, 5.95 mmol) afforded **7**{*5*} (765 mg, 52%) as a yellow oil, after column chromatography (heptane/AcOEt, 3:1°2:1). ^1^H-NMR [400 MHz, δ (ppm), CDCl3]: 6.62 (dd; *J* = 2.7, 1.7 Hz; 1 ^1^H, 5′-C*H*), 6.28 (dd; *J* = 3.6, 1.7 Hz; 1 ^1^H, 3′-C*H*), 6.14 (dd; *J* = 3.6, 2.7 Hz; 1 ^1^H, 4′-C*H*), 4.25 (app t, *J* = 8.2 Hz, 1 ^1^H, 4-C*H*), 3.87 (s, 3 ^1^H, OC*H*_3_), 3.57 (s, 3 ^1^H, 1′-NC*H*_3_), 3.45 (d, *J* = 9.9 Hz, 1 ^1^H, 2-CH*H*), 3.26 (dd; *J* = 9.5, 7.6 Hz; 1 ^1^H, 5-CH*H*), 3.04 (d, *J* = 9.9 Hz, 1 ^1^H, 2-C*H*H), 2.84 (app t, *J* = 9.1 Hz, 1 ^1^H, 5-C*H*H), 2.44 (s, 3 ^1^H, 1-NC*H*_3_). ^13^C-NMR [75 MHz, δ (ppm), CDCl3]: 168.9 (*C*O_2_), 128.2 (2′-*C*), 123.5 (5′-*C*), 117.2 (*C*N), 108.7 (4′-*C*), 107.6 (3′-*C*), 65.2 (2-*C*), 61.3 (5-*C*), 54.2 (O*C*H_3_), 53.8 (3-*C*), 43.7 (4-*C*), 41.5 (1-N*C*H_3_), 34.0 (1′-N*C*H_3_). FTIR [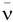 (cm^–1^), neat]: 2951, 2888, 2791, 2242, 1741, 1241, 717. MS [ESI (m/z)] calcd for (C_13_H_17_N_3_O_2_ + H)^+^ = 248, found 248. *R*_F_: 0.23 (heptane/AcOEt, 1:1). Purity: 98.2% (GC).

*(±)-Methyl (3R,4R)-3-cyano-1-methyl-4-(1-methyl-1H-indol-3-yl)pyrrolidine-3-carboxylate* (**7**{*6*}): According to the general procedure, 2-cyanoacrylate **8**{*6*} (9.241 g, 38.46 mmol) afforded **7**{*6*} (10.831 g, 95%) as a yellow solid, after column chromatography (heptane/AcOEt, 3:1°2:1). ^1^H-NMR [400 MHz, δ (ppm), CDCl3]: 7.50 (dt; *J* = 8.1, 1.0 Hz; 1 ^1^H, 4′-C*H*), 7.29 (dt; *J* = 8.3, 1.0 Hz; 1 ^1^H, 7′-C*H*), 7.22 (ddd; *J* = 8.3, 7.1, 1.0 Hz; 1 ^1^H, 6′-C*H*), 7.19 (s, 1 ^1^H, 2′-C*H*), 7.10 (ddd; *J* = 8.1, 7.0, 1.0 Hz; 1 ^1^H, 5′-C*H*), 4.43 (dd; *J* = 9.6, 7.5 Hz; 1 ^1^H, 4-C*H*), 3.76 (s, 3 ^1^H, OC*H*_3_), 3.74 (s, 3 ^1^H, 1′-NC*H*_3_), 3.51 (d, *J* = 10.2 Hz, 1 ^1^H, 2-CH*H*), 3.25 (d, *J* = 10.2 Hz, 1 ^1^H, 2-C*H*H), 3.23 (dd; *J* = 9.6, 7.5 Hz; 1 ^1^H, 5-CH*H*), 3.08 (t, *J* = 9.6 Hz, 1 ^1^H, 5-C*H*H), 2.51 (s, 3 ^1^H, 1-NC*H*_3_). ^13^C-NMR [75 MHz, δ (ppm), CDCl3]: 169.0 (*C*O_2_), 136.8 (7′a-*C*), 127.7 (3′a-*C*), 127.4 (2′-*C*), 122.1 (6′-*C*), 119.5 (5′-*C*), 118.7 (4′-*C*), 118.5 (*C*N), 109.63 (3′-*C*), 109.58 (7′-*C*), 65.3 (2-*C*), 60.9 (5-*C*), 55.1 (3-*C*), 53.9 (O*C*H_3_), 45.2 (4-*C*), 42.0 (1-N*C*H_3_), 33.0 (1′-N*C*H_3_). FTIR [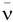 (cm^–1^), neat]: 3045, 2948, 2842, 2786, 2243, 1741, 1474, 1243, 742. HRMS [EI (m/z)] calcd for C_17_H_19_N_3_O_2_ = 297.1477, found for [M^+•^] = 297.1467 (|Δ| = 3.5 ppm), peaks at (relative intensity): 297 (17), 144 (16), 57 (100), 42 (21). *R*_F_: 0.17 (heptane/AcOEt, 1:1). Mp: 92.6 °C. Purity: 98.8% (GC).

*(±)-Methyl (3R,4R)-3-cyano-4- {1-[(dimethylamino)methyl]-1H-indol-3-yl }-1-methylpyrrolidine-3-carboxylate* (**7**{*13*}): According to the general procedure, 2-cyanoacrylate **8**{*8*} (1.512 g, 6.68 mmol) afforded **7**{*13*} (1.929 g, 85%) as a yellow solid. ^1^H-NMR [300 MHz, δ (ppm), CDCl3]: 7.45 (d, *J* = 7.8 Hz, 1 ^1^H, 4′-C*H*), 7.36 (d, *J* = 8.1 Hz, 1 ^1^H, 7′-C*H*), 7.24 (s, 1 ^1^H, 2′-C*H*), 7.14 (ddd; *J* = 8.1, 6.9, 1.0 Hz; 1 ^1^H, 6′-C*H*), 7.05 (ddd; *J* = 7.8, 6.9, 1.0 Hz; 1 ^1^H, 5′-C*H*), 4.69 (d, *J* = 12.9 Hz, 1 ^1^H, NCH*H*N), 4.58 (d, *J* = 12.9 Hz, 1 ^1^H, NC*H*HN), 4.40 (dd; *J* = 9.3, 7.6 Hz; 1 ^1^H, 4-C*H*), 3.67 (s, 3 ^1^H, OC*H*_3_), 3.43 (d, *J* = 10.0 Hz, 1 ^1^H, 2-CH*H*), 3.17 (d, *J* = 10.0 Hz, 1 ^1^H, 2-C*H*H), 3.16 (dd; *J* = 9.3, 7.6 Hz; 1 ^1^H, 5-CH*H*), 3.04 (t, *J* = 9.3 Hz, 1 ^1^H, 5-C*H*H), 2.42 (s, 3 ^1^H, 1-NC*H*_3_), 2.23 (s, 6 ^1^H, N(C*H*_3_)_2_). ^13^C-NMR [75 MHz, δ (ppm), CDCl3]: 168.5 (*C*O_2_), 136.7 (7′a-*C*), 127.5 (3′a-*C*), 126.9 (2′-*C*), 121.9 (6′-*C*), 119.4 (5′-*C*), 118.3 (4′-*C*), 118.0 (*C*N), 110.21 (3′-*C*), 110.17 (7′-*C*), 68.6 (N*C*H_2_N), 65.3 (2-*C*), 60.7 (5-*C*), 55.1 (3-*C*), 53.8 (O*C*H_3_), 44.8 (4-*C*), 42.7 (N(*C*H_3_)_2_), 41.8 (N*C*H_3_). FTIR [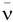 (cm^–1^), neat]: 3049, 2942, 2842, 2778, 2242, 1740, 1460, 1239, 729. Elem. anal. calcd for C_19_H_24_N_4_O_2_: C 67.04%, H 7.11%, N 16.46%; found C 66.98%, H 7.05%, N 16.18%. *R*_F_: 0.19 (AcOEt). Mp: 105.8 °C.

*(±)-Methyl (3R,4R)-3-cyano-1-methyl-4-(2-thienyl)pyrrolidine-3-carboxylate* (**7**{*9*}): According to the general procedure, 2-cyanoacrylate **8**{*9*} (720 mg, 3.73 mmol) afforded **7**{*9*} (814 mg; 87%, combined yield including **11**{*9*}) as a yellow oil, after column chromatography (heptane/AcOEt, 1:1). ^1^H-NMR [300 MHz, δ (ppm), CDCl3]: 7.23 (dd; *J* = 5.1, 1.0 Hz; 1 ^1^H, 5′-C*H*), 7.04 (dt; *J* = 3.6, 1.0 Hz; 1 ^1^H, 3′-C*H*), 6.98 (dd; *J* = 5.1, 3.6 Hz; 1 ^1^H, 4′-C*H*), 4.32 (app t, *J* = 7.8 Hz, 1 ^1^H, 4-C*H*), 3.85 (s, 3 ^1^H, OC*H*_3_), 3.40 (d, *J* = 9.9 Hz, 1 ^1^H, 2-CH*H*), 3.24 (dd; *J* = 9.6, 7.2 Hz; 1 ^1^H, 5-CH*H*), 3.17 (d, *J* = 9.9 Hz, 1 ^1^H, 2-C*H*H), 3.00 (dd; *J* = 9.6, 8.4 Hz; 1 ^1^H, 5-C*H*H), 2.46 (s, 3 ^1^H, NC*H*_3_). MS [ESI (m/z)] calcd for (C_12_H_14_N_2_O_2_S + H)^+^ = 251, found 251. *R*_F_: 0.31 (heptane/AcOEt, 1:1). Purity: 97.6% (GC).

*(±)-Methyl (3R,4R)-3-cyano-1-methyl-4-(2-pyridyl)pyrrolidine-3-carboxylate* (**7**{*10*}): According to the general procedure, 2-cyanoacrylate **8**{*10*} (1.006 g, 5.35 mmol) afforded a 6.5:1 mixture of **7**{*10*}/**11**{*10*} (1.182 g; 90%, combined yield) as a white solid, after column chromatography (CH_2_Cl_2_/MeOH, 24:1). (Measured from a 6.5:1 mixture of **7**{*10*}/**11**{*10*}) ^1^H-NMR [400 MHz, δ (ppm), CDCl3]: 8.61 (d, *J* = 4.8 Hz, 1 ^1^H, 6′-C*H*), 7.68 (app dt; *J* = 1.8, 7.6 Hz; 1 ^1^H, 4′-C*H*), 7.30 (d, *J* = 7.8 Hz, 1 ^1^H, 3′-C*H*), 6.91 (ddd; *J* = 7.6, 4.8, 0.7 Hz; 1 ^1^H, 5′-C*H*), 4.30 (t, *J* = 8.0 Hz, 1 ^1^H, 4-C*H*), 3.88 (s, 3 ^1^H, OC*H*_3_), 3.38 (d, *J* = 9.6 Hz, 1 ^1^H, 2-CH*H*), 3.28 (dd; *J* = 9.3, 8.0 Hz; 1 ^1^H, 5-CH*H*), 3.26 (d, *J* = 9.6 Hz, 1 ^1^H, 2-C*H*H), 3.22 (dd; *J* = 9.3, 8.0 Hz; 1 ^1^H, 5-C*H*H), 2.49 (s, 3 ^1^H, NC*H*_3_). MS [ESI (m/z)] calcd for (C_13_H_15_N_3_O_2_ + H)^+^ = 246, found 246. *R*_F_: 0.07 (heptane/AcOEt, 1:1).

*(±)-Methyl (3R,4R)-3-cyano-1-methyl-4-(3-pyridyl)pyrrolidine-3-carboxylate* (**7**{*11*}): According to the general procedure, 2-cyanoacrylate **8**{*11*} (2.969 g, 15.78 mmol) afforded a 6.6:1 mixture of **7**{*11*}/**11**{*11*} (2.782 g; 72%, combined yield) as a light yellow sticky solid, after column chromatography (CH_2_Cl_2_/MeOH, 29:1). (Measured from a 6.6:1 mixture of **7**{*11*}/**11**{*11*}) ^1^H-NMR [400 MHz, δ (ppm), CDCl3]: 8.56 (dd; *J* = 4.9, 1.7 Hz; 1 ^1^H, 6′-C*H*), 8.56–8.54 (m, 1 ^1^H, 2′-C*H*), 7.81 (app dt; *J* = 8.0, 2.0 Hz; 1 ^1^H, 4′-C*H*), 7.33 (ddd; *J* = 8.0, 4.9, 0.5 Hz; 1 ^1^H, 5′-C*H*), 4.06 (app t, *J* = 7.7 Hz, 1 ^1^H, 4-C*H*), 3.84 (s, 3 ^1^H, OC*H*_3_), 3.32 (d, *J* = 10.0 Hz, 1 ^1^H, 2-CH*H*), 3.28 (d, *J* = 10.0 Hz, 1 ^1^H, 2-C*H*H), 3.09 (dd; *J* = 9.5, 7.8 Hz; 1 ^1^H, 5-CH*H*), 3.06 (dd; *J* = 9.5, 7.6 Hz; 1 ^1^H, 5-C*H*H), 2.47 (s, 3 ^1^H, NC*H*_3_). ^13^C-NMR [75 MHz, δ (ppm), CDCl3]: 168.0 (*C*O_2_), 150.1 (2′-*C*), 149.65 (6′-*C*), 135.9 (4′-*C*), 133.2 (3′-*C*), 123.5 (5′-*C*), 117.6 (*C*N), 65.1 (2-*C*), 60.6 (5-*C*), 54.4 (3-*C*), 54.1 (CO_2_*C*H_3_), 49.4 (4-*C*), 41.3 (N*C*H_3_). MS [ESI (m/z)] calcd for (C_13_H_15_N_3_O_2_ + H)^+^ = 246, found 246. *R*_F_: 0.10 (heptane/AcOEt, 1:1).

*(±)-Methyl (3R,4S)-3-cyano-1-methyl-4-(3-pyridyl)pyrrolidine-3-carboxylate* (**11**{*11*}): According to the general procedure, 2-cyanoacrylate **8**{*11*} (2.969 g, 15.78 mmol) afforded a 6.6:1 mixture of **7**{*11*}/**11**{*11*} (2.782 g; 72%, combined yield) as a light yellow sticky solid, after column chromatography (CH_2_Cl_2_/MeOH, 29:1). (Measured from a 6.6:1 mixture of **7**{*11*}/**11**{*11*}) ^1^H-NMR [400 MHz, δ (ppm), CDCl3]: 8.56–8.54 (m, 1 ^1^H, 2′-C*H*), 8.53 (dd; *J* = 4.9, 1.7 Hz; 1 ^1^H, 6′-C*H*), 7.69 (app dt; *J* = 8.0, 2.0 Hz; 1 ^1^H, 4′-C*H*), 7.25 (ddd; *J* = 8.0, 4.9, 0.5 Hz; 1 ^1^H, 5′-C*H*), 4.07–4.04 (m, 1 ^1^H, 4-C*H*), 3.40 (d, *J* = 10.2 Hz, 1 ^1^H, 2-CH*H*), 3.35 (d, *J* = 10.2 Hz, 1 ^1^H, 2-C*H*H), 3.27 (s, 3 ^1^H, OC*H*_3_), 3.09 (dd; *J* = 9.3, 6.6 Hz; 1 ^1^H, 5-CH*H*), 2.97 (dd; *J* = 9.3, 8.6 Hz; 1 ^1^H, 5-C*H*H), 2.51 (s, 3 ^1^H, NC*H*_3_). ^13^C-NMR [75 MHz, δ (ppm), CDCl3]: 166.6 (*C*O_2_), 150.0 (2′-*C*), 149.60 (6′-*C*), 135.9 (4′-*C*), 131.9 (3′-*C*), 123.2 (5′-*C*), 120.2 (*C*N), 63.4 (2-*C*), 59.6 (5-*C*), 53.4 (4-*C*), 53.1 (CO_2_*C*H_3_), 52.4 (3-*C*), 41.2 (N*C*H_3_). *R*_F_: 0.10 (heptane/AcOEt, 1:1).

*(±)-Methyl (3R,4R)-3-cyano-1-methyl-4-(4-pyridyl)pyrrolidine-3-carboxylate* (**7**{*12*}): According to the general procedure, 2-cyanoacrylate **8**{*12*} (1.037 g, 5.51 mmol) afforded a 5:1 mixture of **7**{*12*}/**11**{*12*} (1.145 g; 85%, combined yield) as a light yellow oil, after column chromatography (CH_2_Cl_2_/MeOH, 24:1). (Measured from a 5:1 mixture of **7**{*12*}/**11**{*12*}) ^1^H-NMR [400 MHz, δ (ppm), CDCl3]: 8.62–8.58 (m, 2 ^1^H, 2′-C*H* + 6′-C*H*), 7.32–7.28 (m, 2 ^1^H, 3′-C*H* + 5′-C*H*), 4.03 (app t, *J* = 7.6 Hz, 1 ^1^H, 4-C*H*), 3.86 (s, 3 ^1^H, OC*H*_3_), 3.32 (d, *J* = 10.0 Hz, 1 ^1^H, 2-CH*H*), 3.26 (d, *J* = 10.0 Hz, 1 ^1^H, 2-C*H*H), 3.09 (dd; *J* = 9.8, 7.2 Hz; 1 ^1^H, 5-CH*H*), 3.06 (dd; *J* = 9.8, 7.8 Hz; 1 ^1^H, 5-C*H*H), 2.47 (s, 3 ^1^H, NC*H*_3_). MS [ESI (m/z)] calcd for (C_13_H_15_N_3_O_2_ + H)^+^ = 246, found 246. *R*_F_: 0.06 (heptane/AcOEt, 1:1).

### 3.4. General procedure for reduction

An excess (7–8 heaped teaspoons) of freshly washed (with MeOH) Raney nickel was added to a solution of α-cyano ester **7** with Et_3_N (ca. 1 equiv) in MeOH (0.25–0.30 M). The mixture was stirred for 15 h at room temperature under a hydrogen atmosphere (1 atm). The catalyst was separated by filtration with suction through a glass filter with a 0.5 cm layer of diatomaceous earth. The catalyst was washed thoroughly with MeOH. The combined methanolic solutions were concentrated on a rotary evaporator.

*(±)-Methyl (3R,4S)-3-(aminomethyl)-1-methyl-4-phenylpyrrolidine-3-carboxylate* (**6**{*1*}): According to the general procedure, α-cyano ester **7**{*1*} (5.040 g, 20.63 mmol) afforded **6**{*1*} (4.858 g, 95%) as a colorless oil. ^1^H-NMR [200 MHz, δ (ppm), CDCl3]: 7.36–7.21 (m, 5 ^1^H, Ph), 3.93 (dd; *J* = 8.6, 7.3 Hz; 1 ^1^H, 4-C*H*), 3.79 (s, 3 ^1^H, OC*H*_3_), 3.35 (d, *J* = 9.6 Hz, 1 ^1^H, 2-CH*H*), 3.08 (dd; *J* = 9.2, 7.3 Hz; 1 ^1^H, 5-CH*H*), 2.77 (app t, *J* = 9.0 Hz, 1 ^1^H, 5-C*H*H), 2.62 (d, *J* = 13.0 Hz, 1 ^1^H, CH*H*NH_2_), 2.52 (d, *J* = 13.0 Hz, 1 ^1^H, C*H*HNH_2_), 2.48 (d, *J* = 9.6 Hz, 1 ^1^H, 2-C*H*H), 2.41 (s, 3 ^1^H, NC*H*_3_), 1.78 (bs, 2 ^1^H, N*H*_2_). ^13^C-NMR [75 MHz, δ (ppm), CDCl3]: 175.9 (*C*O_2_), 138.4 (1′-*C*), 128.7 (2′-*C* + 6′-*C*), 127.9 (3′-*C* + 5′-*C*), 126.6 (4′-*C*), 64.0 (2-*C*), 61.6 (5-*C*), 58.6 (3-*C*), 52.2 (CO_2_*C*H_3_), 49.9 (4-*C*), 46.7 (*C*H_2_NH_2_), 42.2 (N*C*H_3_). FTIR [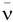 (cm^–1^), neat]: 3386, 3028, 2946, 2781, 1727, 1601, 1455, 1174, 772, 703. HRMS [ESI (m/z)] calcd for (C_14_H_20_N_2_O_2_ + H)^+^ = 249.16030, found 249.15963 (|Δ| = 2.71 ppm). *R*_F_: 0.30 (CH_2_Cl_2_/MeOH, 8:1). Purity: 99.1% (GC).

*(±)-Methyl (3R,4S)-3-(aminomethyl)-4-(4-methoxyphenyl)-1-methylpyrrolidine-3-carboxylate* (**6**{*2*}): According to the general procedure, α-cyano ester **7**{*2*} (6.997 g, 25.51 mmol) afforded **6**{*2*} (6.731 g, 95%) as a colorless oil. ^1^H-NMR [400 MHz, δ (ppm), CDCl3]: 7.27–7.23 (m, 2 ^1^H, 2′-C*H* + 6′-C*H*), 6.86–6.82 (m, 2 ^1^H, 3′-C*H* + 5′-C*H*), 3.88 (app t, *J* = 8.0 Hz, 1 ^1^H, 4-C*H*), 3.79 (s, 3 ^1^H, CO_2_C*H*_3_), 3.78 (s, 3 ^1^H, OC*H*_3_), 3.33 (d, *J* = 9.9 Hz, 1 ^1^H, 2-CH*H*), 3.06 (dd; *J* = 8.8, 7.8 Hz; 1 ^1^H, 5-CH*H*), 2.71 (app t, *J* = 8.9 Hz, 1 ^1^H, 5-C*H*H), 2.63 (d, *J* = 13.2 Hz, 1 ^1^H, CH*H*NH_2_), 2.52 (d, *J* = 13.2 Hz, 1 ^1^H, C*H*HNH_2_), 2.46 (d, *J* = 9.9 Hz, 1 ^1^H, 2-C*H*H), 2.40 (s, 3 ^1^H, NC*H*_3_), 0.92 (bs, 2 ^1^H, N*H*_2_). ^13^C-NMR [75 MHz, δ (ppm), CDCl3]: 176.1 (*C*O_2_), 158.2 (4′-*C*), 130.3 (1′-*C*), 129.7 (2′-*C* + 6′-*C*), 113.5 (3′-*C* + 5′-*C*), 64.1 (2-*C*), 61.8 (5-*C*), 58.6 (3-*C*), 55.2 (O*C*H_3_), 52.3 (CO_2_*C*H_3_), 49.2 (4-*C*), 46.8 (*C*H_2_NH_2_), 42.3 (N*C*H_3_). FTIR [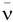 (cm^–1^), neat]: 3383, 3222, 2947, 2834, 2779, 1724, 1247, 834. HRMS [ESI (m/z)] calcd for (C_15_H_22_N_2_O_3_ + H)^+^ = 279.17087, found 279.17006 (|Δ| = 2.87 ppm). *R*_F_: 0.27 (CH_2_Cl_2_/MeOH, 8:1). Purity: 98.1% (GC).

*(±)-Methyl (3R,4S)-3-(aminomethyl)-4-(3,5-dimethoxyphenyl)-1-methylpyrrolidine-3-carboxylate* (**6**{*3*}): According to the general procedure, α-cyano ester **7**{*3*} (7.452 g, 25.06 mmol) afforded **6**{*3*} (6.730 g, 89%) as a yellow oil, after column chromatography (CH_2_Cl_2_/MeOH, 9:1). ^1^H-NMR [400 MHz, δ (ppm), CDCl3]: 6.51 (d, *J* = 2.2 Hz, 2 ^1^H, 2′-C*H* + 6′-C*H*), 6.35 (t, *J* = 2.2 Hz, 1 ^1^H, 4′-C*H*), 3.87 (app t, *J* = 7.9 Hz, 1 ^1^H, 4-C*H*), 3.80 (s, 3 ^1^H, CO_2_C*H*_3_), 3.78 (s, 6 ^1^H, 2 × OC*H*_3_), 3.31 (d, *J* = 9.8 Hz, 1 ^1^H, 2-CH*H*), 3.06 (dd; *J* = 9.0, 7.8 Hz; 1 ^1^H, 5-CH*H*), 2.72 (app t, *J* = 8.8 Hz, 1 ^1^H, 5-C*H*H), 2.71 (d, *J* = 13.0 Hz, 1 ^1^H, CH*H*NH_2_), 2.58 (d, *J* = 13.0 Hz, 1 ^1^H, C*H*HNH_2_), 2.48 (d, *J* = 9.8 Hz, 1 ^1^H, 2-C*H*H), 2.40 (s, 3 ^1^H, NC*H*_3_), 1.45 (bs, 2 ^1^H, N*H*_2_).^ 13^C-NMR [75 MHz, δ (ppm), CDCl3]: 176.5 (*C*O_2_), 160.6 (3′-*C* + 5′-*C*), 141.2 (1′-*C*), 107.5 (2′-*C* + 6′-*C*), 98.6 (4′-*C*), 64.2 (2-*C*), 61.6 (5-*C*), 58.7 (3-*C*), 55.3 (2 × O*C*H_3_), 52.4 (CO_2_*C*H_3_), 50.0 (4-*C*), 46.6 (*C*H_2_NH_2_), 42.3 (N*C*H_3_). FTIR [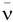 (cm^–1^), neat]: 3380, 3188, 2942, 2835, 2781, 1723, 1593, 1203, 1152. HRMS [ESI (m/z)] calcd for (C_16_H_24_N_2_O_4_ + H)^+^ = 309.18143, found 309.18062 (|Δ| = 2.62 ppm). *R*_F_: 0.30 (CH_2_Cl_2_/MeOH, 8:1). Purity: 98.2% (GC).

*(±)-Methyl (3R,4S)-3-(aminomethyl)-4-(1,3-benzodioxol-5-yl)-1-methylpyrrolidine-3-carboxylate* (**6**{*4*}): According to the general procedure, α-cyano ester **7**{*4*} (7.009 g, 24.31 mmol) afforded **6**{*4*} (6.055 g, 85%) as a light yellow oil, after column chromatography (CH_2_Cl_2_/MeOH, 9:1). ^1^H-NMR [400 MHz, δ (ppm), CDCl3]: 6.88 (d, *J* = 1.7 Hz, 1 ^1^H, 4′-C*H*), 6.78 (dd; *J* = 8.1, 1.5 Hz; 1 ^1^H, 6′-C*H*), 6.72 (d, *J* = 8.1 Hz, 1 ^1^H, 7′-C*H*), 5.92 (s, 2 ^1^H, 2′-C*H*_2_), 3.83 (app t, *J* = 7.7 Hz, 1 ^1^H, 4-C*H*), 3.78 (s, 3 ^1^H, OC*H*_3_), 3.27 (d, *J* = 9.9 Hz, 1 ^1^H, 2-CH*H*), 3.01 (dd; *J* = 9.3, 7.6 Hz; 1 ^1^H, 5-CH*H*), 2.67 (app t, *J* = 8.7 Hz, 1 ^1^H, 5-C*H*H), 2.66 (d, *J* = 12.9 Hz, 1 ^1^H, CH*H*NH_2_), 2.53 (d, *J* = 12.9 Hz, 1 ^1^H, C*H*HNH_2_), 2.48 (d, *J* = 9.9 Hz, 1 ^1^H, 2-C*H*H), 2.38 (s, 3 ^1^H, NC*H*_3_), 1.01 (bs, 2 ^1^H, N*H*_2_). ^13^C-NMR [75 MHz, δ (ppm), CDCl3]: 176.3 (*C*O_2_), 147.4 (3′a-*C*), 146.3 (7′a-*C*), 132.5 (5′-*C*), 122.0 (6′-*C*), 109.3 (4′-*C*), 107.8 (7′-*C*), 100.8 (2′-*C*), 63.8 (2-*C*), 61.8 (5-*C*), 58.6 (3-*C*), 52.1 (O*C*H_3_), 49.5 (4-*C*), 46.6 (*C*H_2_NH_2_), 42.0 (N*C*H_3_). FTIR [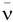 (cm^–1^), neat]: 3320, 3317, 2946, 2841, 2776, 1721, 1248, 1232, 1034, 929. HRMS [ESI (m/z)] calcd for (C_15_H_20_N_2_O_4_ + H)^+^ = 293.15013, found 293.14936 (|Δ| = 2.63 ppm). *R*_F_: 0.25 (CH_2_Cl_2_/MeOH, 8:1). Purity: 97.4% (GC).

*(±)-Methyl (3R,4S)-3-(aminomethyl)-1-methyl-4-(1-methyl-1H-pyrrol-2-yl)pyrrolidine-3-carboxylate* (**6**{*5*}): According to the general procedure, α-cyano ester **7**{*5*} (752 mg, 3.04 mmol) afforded **6**{*5*} (559 mg, 73%) as a light yellow oil, after column chromatography (CH_2_Cl_2_/MeOH, 9:1). ^1^H-NMR [400 MHz, δ (ppm), CDCl3]: 6.54 (dd; *J* = 2.4, 1.8 Hz; 1 ^1^H, 5′-C*H*), 6.07 (dd; *J* = 3.6, 2.7 Hz; 1 ^1^H, 4′-C*H*), 6.04 (dd; *J* = 3.8, 1.7 Hz; 1 ^1^H, 3′-C*H*), 4.10 (dd; *J* = 10.5, 7.2 Hz; 1 ^1^H, 4-C*H*), 3.78 (s, 3 ^1^H, OC*H*_3_), 3.60 (s, 3 ^1^H, 1′-NC*H*_3_), 3.39 (d, *J* = 9.9 Hz, 1 ^1^H, 2-CH*H*), 3.19 (app t, *J* = 8.2 Hz, 1 ^1^H, 5-CH*H*), 2.72 (d, *J* = 13.2 Hz, 1 ^1^H, CH*H*NH_2_), 2.62 (d, *J* = 13.2 Hz, 1 ^1^H, C*H*HNH_2_), 2.47 (app t, *J* = 9.8 Hz, 1 ^1^H, 5-C*H*H), 2.35 (s, 3 ^1^H, 1-NC*H*_3_), 2.21 (d, *J* = 9.9 Hz, 1 ^1^H, 2-C*H*H), 1.35 (bs, 2 ^1^H, N*H*_2_). ^13^C-NMR [75 MHz, δ (ppm), CDCl3]: 177.1 (*C*O_2_), 129.8 (2′-*C*), 122.3 (5′-*C*), 107.8 (3′-*C*), 107.3 (4′-*C*), 65.3 (2-*C*), 62.5 (5-*C*), 58.4 (3-*C*), 52.5 (O*C*H_3_), 46.7 (*C*H_2_NH_2_), 42.2 (1-N*C*H_3_), 40.9 (4-*C*), 34.2 (1′-N*C*H_3_). FTIR [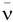 (cm^–1^), neat]: 3390, 3099, 2946, 2839, 2780, 1729, 1242, 712. HRMS [ESI (m/z)] calcd for (C_13_H_21_N_3_O_2_ + H)^+^ = 252.17120, found 252.17083 (|Δ| = 1.49 ppm). *R*_F_: 0.24 (CH_2_Cl_2_/MeOH, 8:1). Purity: 97.7% (GC).

*(±)-Methyl (3R,4S)-3-(aminomethyl)-1-methyl-4-(1-methyl-1H-indol-3-yl)pyrrolidine-3-carboxylate* (**6**{*6*}): According to the general procedure, α-cyano ester **7**{*6*} (7.628 g, 25.65 mmol) afforded **6**{*6*} (7.337 g, 95%) as a yellow oil, after column chromatography (CH_2_Cl_2_/MeOH, 9:1). ^1^H-NMR [400 MHz, δ (ppm), CDCl3]: 7.72 (dt; *J* = 8.0, 1.0 Hz; 1 ^1^H, 4′-C*H*), 7.28 (dt; *J* = 8.0, 1.0 Hz; 1 ^1^H, 7′-C*H*), 7.21 (ddd; *J* = 8.0, 6.8, 1.0 Hz; 1 ^1^H, 6′-C*H*), 7.11 (ddd; *J* = 8.0, 6.8, 1.0 Hz; 1 ^1^H, 5′-C*H*), 6.98 (s, 1 ^1^H, 2′-C*H*), 4.32 (dd; *J* = 9.3, 7.3 Hz; 1 ^1^H, 4-C*H*), 3.83 (s, 3 ^1^H, OC*H*_3_), 3.76 (s, 3 ^1^H, 1′-NC*H*_3_), 3.43 (d, *J* = 10.0 Hz, 1 ^1^H, 2-CH*H*), 3.17 (dd; *J* = 9.3, 7.3 Hz; 1 ^1^H, 5-CH*H*), 2.75 (d, *J* = 13.2 Hz, 1 ^1^H, CH*H*NH_2_), 2.68 (t, *J* = 9.3 Hz, 1 ^1^H, 5-C*H*H), 2.60 (d, *J* = 13.2 Hz, 1 ^1^H, C*H*HNH_2_), 2.45 (d, *J* = 10.0 Hz, 1 ^1^H, 2-C*H*H), 2.42 (s, 3 ^1^H, 1-NC*H*_3_), 1.04 (bs, 2 ^1^H, N*H*_2_). ^13^C-NMR [75 MHz, δ (ppm), CDCl3]: 177.1 (*C*O_2_), 136.9 (7′a-*C*), 128.3 (3′a-*C*), 127.3 (2′-*C*), 122.0 (6′-*C*), 119.9 (5′-*C*), 119.3 (4′-*C*), 112.0 (3′-*C*), 109.3 (7′-*C*), 64.8 (2-*C*), 62.5 (5-*C*), 58.4 (3-*C*), 52.4 (O*C*H_3_), 47.1 (*C*H_2_NH_2_), 42.5 (4-*C*), 41.6 (1-N*C*H_3_), 32.9 (1′-N*C*H_3_). FTIR [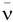 (cm^–1^), neat]: 3386, 3047, 2946, 2834, 2779, 1720, 1472, 1172, 741. HRMS [EI (m/z)] calcd for C_17_H_23_N_3_O_2_ = 301.1790, found for [M^+•^] = 301.1776 (|Δ| = 4.7 ppm), peaks at (relative intensity): 301 (7), 284 (93), 228 (18), 157 (100), 144 (22), 57 (27). *R*_F_: 0.22 (CH_2_Cl_2_/MeOH, 8:1). Purity: 98.0% (GC).

*(±)-(3R,4R)-3-Cyano-1-methyl-4-phenylpyrrolidine-3-carboxamide* (**12**{*1*}): ^1^H-NMR [300 MHz, δ (ppm), CDCl3]: 7.41–7.26 (m, 5 ^1^H, Ph), 6.58 (s, 1 ^1^H, NH*H*), 6.40 (s, 1 ^1^H, N*H*H), 3.99 (app t, *J* = 8.1 Hz, 1 ^1^H, 4-C*H*), 3.34 (d, *J* = 9.8 Hz, 1 ^1^H, 2-CH*H*), 3.16 (app t, *J* = 8.7 Hz, 1 ^1^H, 5-CH*H*), 3.12 (d, *J* = 9.8 Hz, 1 ^1^H, 2-C*H*H), 3.03 (app t, *J* = 8.9 Hz, 1 ^1^H, 5-C*H*H), 2.47 (s, 3 ^1^H, NC*H*_3_). ^13^C-NMR [75 MHz, δ (ppm), CDCl3]: 169.4 (*C*ON), 137.1 (1′-*C*), 128.6 (2′-*C* + 6′-*C*), 128.4 (3′-*C* + 5′-*C*), 128.2 (4′-*C*), 119.0 (*C*N), 65.2 (2-*C*), 60.9 (5-*C*), 55.9 (3-*C*), 53.1 (4-*C*), 41.6 (N*C*H_3_). FTIR [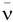 (cm^–1^), neat]: 3334, 3192, 2950, 2846, 2794, 2241, 1684, 772, 698. Elem. anal. calcd for C_13_H_15_N_3_O: C 68.11%, H 6.59%, N 18.33%; found C 68.10%, H 6.48%, N 18.13%. *R*_F_: 0.40 (AcOEt). Mp: 121.7 °C (from propan-2-ol, small white needles). Purity: >99.5% (GC).

### 3.5. Parallel synthesis

#### 3.5.1. General procedure 1 for spiro dihydrouracil/α-ureidomethyl acid formation using parallel synthesis

A solution of isocyanate **13** (0.12 mmol for **1**, 0.10 mmol for **3**–**5**) from a 0.3 M stock solution in CH_2_Cl_2_ was added to a solution of α-aminomethyl ester (0.10 mmol) in CH_2_Cl_2_ (1.5 mL). The resulting reaction mixture was stirred at room temperature for 15 h. After that time, the solvent was evaporated and THF (1.5 mL) and 1 M KOBu*^t^* in THF (0.10 mmol) were added. The reaction mixture was then stirred at room temperature for 15 h. A saturated solution of NH_4_Cl (1.0 mL) was added and the layers were separated (centrifugation was needed for the separation when aryl isocyanate was used). The aqueous layer was extracted with CH_2_Cl_2_ (2 × 1.5 mL) and the combined organic layers were evaporated to dryness under vacuum.

#### 3.5.2. General procedure 2 for spiro dihydrouracil/α-ureidomethyl acid formation using parallel synthesis

A solution of isocyanate **13**{*2*,*6*–*8*} (0.10 mmol) from a 0.3 M stock solution in DMF was added to a solution of α-aminomethyl ester (0.10 mmol) in DMF (1.5 mL). The resulting reaction mixture was stirred at 80 °C for 15 h. After that time, the solvent was evaporated and THF (1.5 mL) and 1 M KOBu*^t^* in THF (0.10 mmol) were added. The reaction mixture was then stirred at room temperature for 15 h. A saturated solution of NH_4_Cl (1.0 mL) was added and the layers were separated (centrifugation was needed for the separation when aryl isocyanate was used). The aqueous layer was extracted with CH_2_Cl_2_ (2 × 1.5 mL) and the combined organic layers were evaporated to dryness under vacuum.

*(±)-(4R,5S)-7-Ethyl-2-methyl-4-phenyl-2,7,9-triazaspiro[4,5]decane-6,8-dione* (**5**{*1*,*1*}): (From **6**{*1*}) ^1^H-NMR [400 MHz, δ (ppm), CDCl3]: 7.30–7.19 (m, 5 ^1^H, Ph), 5.60 (bd, *J* = 2.1 Hz, 1 ^1^H, N*H*), 4.20 (dd; *J* = 8.1, 5.4 Hz; 1 ^1^H, 4-C*H*), 3.85 (dq; *J* = 12.9, 7.2 Hz; 1 ^1^H, CH*H*CH_3_), 3.80 (dq; *J* = 12.9, 7.2 Hz; 1 ^1^H, C*H*HCH_3_), 3.06 (dd; *J* = 9.6, 5.4 Hz; 1 ^1^H, 3-CH*H*), 2.99 (dd; *J* = 9.6, 8.1 Hz; 1 ^1^H, 3-C*H*H), 2.95 (d, *J* = 9.3 Hz, 1 ^1^H, 1-CH*H*), 2.94 (dd; *J* = 12.6, 4.2 Hz; 1 ^1^H, 10-CH*H*), 2.68 (d, *J* = 9.3 Hz, 1 ^1^H, 1-C*H*H), 2.66 (d, *J* = 12.6 Hz, 1 ^1^H, 10-C*H*H), 2.41 (s, 3 ^1^H, NC*H*_3_), 1.16 (t, *J* = 7.2 Hz, 3 ^1^H, CH_2_C*H*_3_). ^13^C-NMR [75 MHz, δ (ppm), CDCl3]: 173.1 (6-*C*), 154.1 (8-*C*), 139.4 (1′-*C*), 128.7 (2′-*C* + 6′-*C*), 128.6 (3′-*C* + 5′-*C*), 127.3 (4′-*C*), 64.0 (1-*C*), 62.0 (3-*C*), 52.0 (5-*C*), 48.3 (4-*C*), 43.4 (10-*C*), 42.0 (N*C*H_3_), 36.4 (*C*H_2_CH_3_), 13.8 (CH_2_*C*H_3_). FTIR [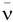 (cm^–1^), neat]: 3240, 2937, 2841, 2784, 1716, 1673, 763, 703. MS [APCI (m/z)] calcd for (C_16_H_21_N_3_O_2_ + H)^+^ = 288, found 288. Crude yield: 51%. Purity: 89% (LC).

*(±)-(4R,5S)-2-Methyl-7-phenethyl-4-phenyl-2,7,9-triazaspiro[4,5] decane-6,8-dione* (**5**{*1*,*2*}): (From **6**{*1*}) ^1^H-NMR [400 MHz, δ (ppm), CDCl3]: 7.33–7.15 (m, 10 ^1^H, 2 × Ph), 5.66 (bd, *J* = 3.4 Hz, 1 ^1^H, N*H*), 4.16 (dd; *J* = 8.0, 5.4 Hz; 1 ^1^H, 4-C*H*), 4.04 (dt; *J* = 13.0, 7.6 Hz; 1 ^1^H, NCH*H*CH_2_), 3.98 (dt; *J* = 13.0, 7.6 Hz; 1 ^1^H, NC*H*HCH_2_), 3.05 (dd; *J* = 9.3, 5.4 Hz; 1 ^1^H, 3-CH*H*), 2.98–2.83 (m, 5 ^1^H, 1-CH*H* + 3-C*H*H + 10-CH*H* + NCH_2_C*H*_2_), 2.56 (d, *J* = 9.5 Hz, 1 ^1^H, 1-C*H*H), 2.55 (d, *J* = 13.2 Hz, 1 ^1^H, 10-C*H*H), 2.39 (s, 3 ^1^H, NC*H*_3_). ^13^C-NMR [75 MHz, δ (ppm), CDCl3]: 173.2 (6-*C*), 154.0 (8-*C*), 139.3 (1′′-*C*), 138.6 (1′-*C*), 129.2 (2′-*C* + 6′-*C*), 128.8 (2′′-*C* + 6′′-*C*), 128.6 (3′′-*C* + 5′′-*C*), 128.5 (3′-*C* + 5′-*C*), 127.3 (4′′-*C*), 126.5 (4′-*C*), 64.0 (1-*C*), 61.9 (3-*C*), 52.1 (5-*C*), 48.2 (4-*C*), 43.4 (10-*C*), 42.2 (N*C*H_2_CH_2_), 41.9 (N*C*H_3_), 34.5 (NCH_2_*C*H_2_). FTIR [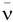 (cm^–1^), neat]: 3254, 2938, 2841, 2784, 1717, 1673, 758, 701. MS [APCI (m/z)] calcd for (C_22_H_25_N_3_O_2_ + H)^+^ = 364, found 364. Crude yield: 63%. Purity: 70% (LC).

*(±)-Ethyl 3-{(4R,5S)-2-methyl-6,8-dioxo-4-phenyl-2,7,9-triazaspiro[4,5] decan-7-yl}propanoate* (**5**{*1*,*3*}): (From **6**{*1*}) MS [APCI (m/z)] calcd for (C_19_H_25_N_3_O_4_ + H)^+^ = 360, found 360. Crude yield: 80% (combined yield of **5**{*1*,*3*}, **5**{*1*,*9*}, **5**{*1*,*10*}, and **5**{*1*,*11*}). Purity: 91% (LC; combined purity of **5**{*1*,*3*}, **5**{*1*,*9*}, **5**{*1*,*10*}, and **5**{*1*,*11*}).

*(±)-(4R,5S)-7-Ethyl-4-(4-methoxyphenyl)-2-methyl-2,7,9-triazaspiro[4,5] decane-6,8-dione* (**5**{*2*,*1*}): (From **6**{*2*}) MS [APCI (m/z)] calcd for (C_17_H_23_N_3_O_3_ + H)^+^ = 318, found 318. Crude yield: 60%. Purity: 93% (LC).

*(±)-(4R,5S)-4-(4-Methoxyphenyl)-2-methyl-7-phenethyl-2,7,9-triazaspiro[4,5] decane-6,8-dione* (**5**{*2*,*2*}): (From **6**{*2*}) MS [APCI (m/z)] calcd for (C_23_H_27_N_3_O_3_ + H)^+^ = 394, found 394. Crude yield: 64%. Purity: 60% (LC).

*(±)-Ethyl 3-{(4R,5S)-4-(4-methoxyphenyl)-2-methyl-6,8-dioxo-2,7,9-triazaspiro[4,5] decan-7-yl}prop-anoate* (**5**{*2*,*3*}): (From **6**{*2*}) FTIR [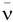 (cm^–1^), neat]: 3245, 2935, 2837, 2784, 1719, 1674, 1247, 835. MS [APCI (m/z)] calcd for (C_20_H_27_N_3_O_5_ + H)^+^ = 390, found 390. Crude yield: 49% (combined yield of **5**{*2*,*3*}, **5**{*2*,*9*}, **5**{*2*,*10*}, and **5**{*2*,*11*}). Purity: 88% (LC; combined purity of **5**{*2*,*3*}, **5**{*2*,*9*}, **5**{*2*,*10*}, and **5**{*2*,*11*}).

*(±)-(4R,5S)-4-(3,5-Dimethoxyphenyl)-7-ethyl-2-methyl-2,7,9-triazaspiro[4,5] decane-6,8-dione*** 5**{*3*,*1*}): (From **6**{*3*}) MS [APCI (m/z)] calcd for (C_18_H_25_N_3_O_4_ + H)^+^ = 348, found 348. Crude yield: 66%. Purity: 90% (LC).

*(±)-(4R,5S)-4-(3,5-Dimethoxyphenyl)-2-methyl-7-phenethyl-2,7,9-triazaspiro[4,5] decane-6,8-dione*

**5**{*3*,*2*}): (From **6**{*3*}) MS [APCI (m/z)] calcd for (C_24_H_29_N_3_O_4_ + H)^+^ = 424, found 424. Crude yield: 60%. Purity: 68% (LC).

*(±)-Ethyl 3-{(4R,5S)-4-(3,5-dimethoxyphenyl)-2-methyl-6,8-dioxo-2,7,9-triazaspiro[4,5] decan-7-yl}-propanoate* (**5**{*3*,*3*}): (From **6**{*3*}) MS [APCI (m/z)] calcd for (C_21_H_29_N_3_O_6_ + H)^+^ = 420, found 420. Crude yield: 54% (combined yield of **5**{*3*,*3*}, **5**{*3*,*9*}, **5**{*3*,*10*}, and **5**{*3*,*11*}). Purity: 95% (LC; combined purity of **5**{*3*,*3*}, **5**{*3*,*9*}, **5**{*3*,*10*}, and **5**{*3*,*11*}).

*(±)-(4R,5S)-4-(1,3-Benzodioxol-5-yl)-7-ethyl-2-methyl-2,7,9-triazaspiro[4,5] decane-6,8-dione*

(**5**{*4*,*1*}): (From **6**{*4*}) MS [APCI (m/z)] calcd for (C_17_H_21_N_3_O_4_ + H)^+^ = 332, found 332. Crude yield: 63%. Purity: 95% (LC).

*(±)-(4R,5S)-4-(1,3-Benzodioxol-5-yl)-2-methyl-7-phenethyl-2,7,9-triazaspiro[4,5]* *decane-6,8-dione*

**5**{*4*,*2*}): (From **6**{*4*}) MS [APCI (m/z)] calcd for (C_23_H_25_N_3_O_4_ + H)^+^ = 408, found 408. Crude yield: 64%. Purity: 68% (LC).

*(±)-Ethyl 3-{(4R,5S)-4-(1,3-benzodioxol-5-yl)-2-methyl-6,8-dioxo-2,7,9-triazaspiro[4,5]decan-7-yl}-propanoate* (**5**{*4*,*3*}): (From **6**{*4*}) MS [APCI (m/z)] calcd for (C_20_H_25_N_3_O_6_ + H)^+^ = 404, found 404. Crude yield: 61% (combined yield of **5**{*4*,*3*}, **5**{*4*,*9*}, **5**{*4*,*10*}, and **5**{*4*,*11*}). Purity: 98% (LC; combined purity of **5**{*4*,*3*}, **5**{*4*,*9*}, **5**{*4*,*10*}, and **5**{*4*,*11*}).

*(±)-(4R,5S)-7-Ethyl-2-methyl-4-(1-methyl-1H-pyrrol-2-yl)-2,7,9-triazaspiro[4,5] decane-6,8-dione*

(**5**{*5*,*1*}): (From **6**{*5*}) ^1^H-NMR [400 MHz, δ (ppm), CDCl3]: 6.52 (dd; *J* = 2.8, 1.6 Hz; 1 ^1^H, 5′-C*H*), 6.05 (dd; *J* = 3.5, 2.8 Hz; 1 ^1^H, 4′-C*H*), 6.03 (dd; *J* = 3.5, 1.6 Hz; 1 ^1^H, 3′-C*H*), 5.84 (bd, *J* = 3.5 Hz, 1 ^1^H, N*H*), 4.35 (app t, *J* = 8.0 Hz, 1 ^1^H, 4-C*H*), 3.85 (dq; *J* = 13.1, 7.0 Hz; 1 ^1^H, CH*H*CH_3_), 3.79 (dq; *J* = 13.1, 7.0 Hz; 1 ^1^H, C*H*HCH_3_), 3.44 (s, 3 ^1^H, 1′-NC*H*_3_), 3.15 (app t, *J* = 8.6 Hz, 1 ^1^H, 3-CH*H*), 2.96 (dd; *J* = 13.0, 4.6 Hz; 1 ^1^H, 10-CH*H*), 2.92 (d, *J* = 9.8 Hz, 1 ^1^H, 1-CH*H*), 2.78 (app t, *J* = 8.8 Hz, 1 ^1^H, 3-C*H*H), 2.65 (d, *J* = 9.8 Hz, 1 ^1^H, 1-C*H*H), 2.62 (d, *J* = 13.0 Hz, 1 ^1^H, 10-C*H*H), 2.37 (s, 3 ^1^H, 2-NC*H*_3_), 1.15 (t, *J* = 7.0 Hz, 3 ^1^H, CH_2_C*H*_3_). ^13^C-NMR [75 MHz, δ (ppm), CDCl3]: 173.5 (6-*C*), 154.2 (8-*C*), 129.9 (2′-*C*), 122.4 (5′-*C*), 107.5 (3′-*C*), 107.2 (4′-*C*), 66.0 (1-*C*), 62.0 (3-*C*), 51.0 (5-*C*), 43.9 (10-*C*), 41.9 (2-N*C*H_3_), 39.5 (4-*C*), 36.4 (*C*H_2_CH_3_), 34.1 (1′-N*C*H_3_), 13.7 (CH_2_*C*H_3_). FTIR [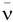 (cm^–1^), neat]: 3329, 2937, 2842, 2784, 1716, 1671, 727. MS [APCI (m/z)] calcd for (C_15_H_22_N_4_O_2_ + H)^+^ = 291, found 291. Crude yield: 61%. Purity: 99% (LC).

*(±)-(4R,5S)-2-Methyl-4-(1-methyl-1H-pyrrol-2-yl)-7-phenethyl-2,7,9-triazaspiro[4,5] decane-6,8-dione* (**5**{*5*,*2*}): (From **6**{*5*}) ^1^H-NMR [400 MHz, δ (ppm), CDCl3]: 7.32–7.15 (m, 5 ^1^H, Ph), 6.51 (dd; *J* = 2.8, 1.6 Hz; 1 ^1^H, 5′-C*H*), 6.05 (dd; *J* = 3.5, 2.8 Hz; 1 ^1^H, 4′-C*H*), 6.02 (dd; *J* = 3.5, 1.6 Hz; 1 ^1^H, 3′-C*H*), 5.96 (bd, *J* = 4.0 Hz, 1 ^1^H, N*H*), 4.31 (app t, *J* = 8.1 Hz, 1 ^1^H, 4-C*H*), 4.08–3.95 (m, 2 ^1^H, NC*H*_2_CH_2_), 3.34 (s, 3 ^1^H, 1′-NC*H*_3_), 3.13 (app t, *J* = 8.6 Hz, 1 ^1^H, 3-CH*H*), 2.92 (dd; *J* = 12.9, 4.7 Hz; 1 ^1^H, 10-CH*H*), 2.91–2.83 (m, 2 ^1^H, NCH_2_C*H*_2_), 2.80 (d, *J* = 9.8 Hz, 1 ^1^H, 1-CH*H*), 2.76 (app t, *J* = 8.8 Hz, 1 ^1^H, 3-C*H*H), 2.62 (d, *J* = 9.8 Hz, 1 ^1^H, 1-C*H*H), 2.55 (d, *J* = 12.9 Hz, 1 ^1^H, 10-C*H*H), 2.35 (s, 3 ^1^H, 2-NC*H*_3_). ^13^C-NMR [75 MHz, δ (ppm), CDCl3]: 173.6 (6-*C*), 154.2 (8-*C*), 138.6 (1′′-*C*), 129.8 (2′-*C*), 129.2 (2′′-*C* + 6′′-*C*), 128.5 (3′′-*C* + 5′′-*C*), 126.5 (4′′-*C*), 122.4 (5′-*C*), 107.5 (3′-*C*), 107.2 (4′-*C*), 65.9 (1-*C*), 62.0 (3-*C*), 51.0 (5-*C*), 43.8 (10-*C*), 42.2 (N*C*H_2_CH_2_), 41.9 (2-N*C*H_3_), 39.3 (4-*C*), 34.4 (NCH_2_*C*H_2_), 34.1 (1′-N*C*H_3_). FTIR [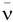 (cm^–1^), neat]: 3251, 2941, 2843, 2785, 1716, 1671, 761, 728, 699. MS [APCI (m/z)] calcd for (C_21_H_26_N_4_O_2_ + H)^+^ = 367, found 367. Crude yield: 54%. Purity: 92% (LC).

*(±)-Ethyl 3-{(4R,5S)-2-methyl-4-(1-methyl-1H-pyrrol-2-yl)-6,8-dioxo-2,7,9-triazaspiro[4,5] decan-7-yl}propanoate* (**5**{*5*,*3*}): (From **6**{*5*}) MS [APCI (m/z)] calcd for (C_18_H_26_N_4_O_4_ + H)^+^ = 363, found 363. Crude yield: 66% (combined yield of **5**{*5*,*3*}, **5**{*5*,*9*}, **5**{*5*,*10*}, and **5**{*5*,*11*}). Purity: 79% (LC; combined purity of **5**{*5*,*3*}, **5**{*5*,*9*}, **5**{*5*,*10*}, and **5**{*5*,*11*}).

*(±)-(4R,5S)-7-Ethyl-2-methyl-4-(1-methyl-1H-indol-3-yl)-2,7,9-triazaspiro[4,5] decane-6,8-dione*

(**5**{*6*,*1*}): (From **6**{*6*}) MS [APCI (m/z)] calcd for (C_19_H_24_N_4_O_2_ + H)^+^ = 341, found 341. Crude yield: 69%. Purity: 69% (LC).

*(±)-(4R,5S)-2-Methyl-4-(1-methyl-1H-indol-3-yl)-7-phenethyl-2,7,9-triazaspiro[4,5] decane-6,8-dione*

(**5**{*6*,*2*}): (From **6**{6}) MS [APCI (m/z)] calcd for (C_25_H_28_N_4_O_2_ + H)+ = 417, found 417. Crude yield: 68%. Purity: 62% (LC).

*(±)-Ethyl 3-{(4R,5S)-2-methyl-4-(1-methyl-1H-indol-3-yl)-6,8-dioxo-2,7,9-triazaspiro[4,5] decan-7-yl}-propanoate* (**5**{*6*,*3*}): (From **6**{*6*}) MS [APCI (m/z)] calcd for (C_22_H_28_N_4_O_4_ + H)^+^ = 413, found 413. Crude yield: 62% (combined yield of **5**{*6*,*3*}, **5**{*6*,*9*}, **5**{*6*,*10*}, and **5**{*6*,*11*}). Purity: 86% (LC; combined purity of **5**{*6*,*3*}, **5**{*6*,*9*}, **5**{*6*,*10*}, and **5**{*6*,*11*}).

*(±)-(3R,4S)-1-Methyl-4-phenyl-3-[(3-phenylureido)methyl]pyrrolidine-3-carboxylic acid* (**14**{*1*,*4*}): (From **6**{*1*}) MS [APCI (m/z)] calcd for (C_20_H_23_N_3_O_3_ + H)^+^ = 354, found 354. Crude yield: 49%. Purity: >99% (LC).

*(±)-(3R,4S)-1-Methyl-4-phenyl-3-({3-[4-(trifluoromethyl)phenyl]ureido}methyl)pyrrolidine-3-carboxy-lic acid* (**14**{*1*,*5*}): (From **6**{*1*}) MS [APCI (m/z)] calcd for (C_21_H_22_F_3_N_3_O_3_ + H)^+^ = 422, found 422. Crude yield: 75%. Purity: 97% (LC).

*(±)-(3R,4S)-3-{[3-(4-Ethoxyphenyl)ureido]methyl}-1-methyl-4-phenylpyrrolidine-3-carboxylic acid* (**14**{*1*,*6*}): (From **6**{*1*}) MS [APCI (m/z)] calcd for (C_22_H_27_N_3_O_4_ + H)^+^ = 398, found 398. Crude yield: 48%. Purity: >99% (LC).

*(±)-(3R,4S)-3-{[3-(3-Cyanophenyl)ureido]methyl}-1-methyl-4-phenylpyrrolidine-3-carboxylic acid* (**14**{*1*,*7*}): (From **6**{*1*}) MS [APCI (m/z)] calcd for (C_21_H_22_N_4_O_3_ + H)^+^ = 379, found 379. Crude yield: 54%. Purity: >99% (LC).

*(±)-(3R,4S)-1-Methyl-4-phenyl-3-{[3-(3-pyridyl)ureido]methyl}pyrrolidine-3-carboxylic acid* (**14**{*1*,*8*}): (From **6**{*1*}) MS [APCI (m/z)] calcd for (C_19_H_22_N_4_O_3_ + H)^+^ = 355, found 355. Crude yield: 49%. Purity: 79% (LC).

*(±)-(3R,4S)-4-(4-Methoxyphenyl)-1-methyl-3-[(3-phenylureido)methyl]pyrrolidine-3-carboxylic acid* (**14**{*2*,*4*}): (From **6**{*2*}) MS [APCI (m/z)] calcd for (C_21_H_25_N_3_O_4_ + H)^+^ = 384, found 384. Crude yield: 60%. Purity: 99% (LC).

*(±)-(3R,4S)-4-(4-Methoxyphenyl)-1-methyl-3-({3-[4-(trifluoromethyl)phenyl]ureido}methyl)-pyrroli-dine-3-carboxylic acid* (**14**{*2*,*5*}): (From **6**{*2*}) MS [APCI (m/z)] calcd for (C_22_H_24_F_3_N_3_O_4_ + H)^+^ = 452, found 452. Crude yield: 65%. Purity: 98% (LC).

*(±)-(3R,4S)-3-{[3-(4-Ethoxyphenyl)ureido]* *methyl}-4-(4-methoxyphenyl)-1-methylpyrrolidine-3-car-*

*boxylic acid* (**14**{*2*,*6*}): (From **6**{*2*}) MS [APCI (m/z)] calcd for (C_23_H_29_N_3_O_5_ + H)^+^ = 428, found 428. Crude yield: 70%. Purity: >99% (LC).

*(±)-(3R,4S)-3-{[3-(3-Cyanophenyl)ureido]* *methyl}-4-(4-methoxyphenyl)-1-methylpyrrolidine-3-car-*

*boxylic acid* (**14**{*2*,*7*}): (From **6**{*2*}) MS [APCI (m/z)] calcd for (C_22_H_24_N_4_O_4_ + H)^+^ = 409, found 409. Crude yield: 60%. Purity: 96% (LC).

*(±)-(3R,4S)-4-(4-Methoxyphenyl)-1-methyl-3-{[3-(3-pyridyl)ureido]* *methyl}pyrrolidine-3-carboxylic*

* acid* (**14**{*2*,*8*}): (From **6**{*2*}) MS [APCI (m/z)] calcd for (C_20_H_24_N_4_O_4_ + H)^+^ = 385, found 385. Crude yield: 45%. Purity: 76% (LC).

*(±)-(3R,4S)-4-(3,5-Dimethoxyphenyl)-1-methyl-3-[(3-phenylureido)methyl]pyrrolidine-3-carboxylic acid* (**14**{*3*,*4*}): (From **6**{*3*}) MS [APCI (m/z)] calcd for (C_22_H_27_N_3_O_5_ + H)^+^ = 414, found 414. Crude yield: 76%. Purity: 97% (LC).

*(±)-(3R,4S)-4-(3,5-Dimethoxyphenyl)-1-methyl-3-({3-[4-(trifluoromethyl)phenyl]ureido}methyl)pyrro-lidine-3-carboxylic acid* (**14**{*3*,*5*}): (From **6**{*3*}) MS [APCI (m/z)] calcd for (C_23_H_26_F_3_N_3_O_5_ + H)^+^ = 482, found 482. Crude yield: 63%. Purity: 99% (LC).

*(±)-(3R,4S)-4-(3,5-Dimethoxyphenyl)-3-{[3-(4-Ethoxyphenyl)ureido]* *methyl}-1-methylpyrrolidine-3-*

*carboxylic acid* (**14**{*3*,*6*}): (From **6**{*3*}) ^1^H-NMR [300 MHz, δ (ppm), CD3SOCD3]: 8.83 (bs, 1 ^1^H, N*H*Ar), 7.30–7.21 (m, 2 ^1^H, 2′′-C*H* + 6′′-C*H*), 6.79–6.70 (m, 2 ^1^H, 3′′-C*H* + 5′′-C*H*), 6.47 (d, *J* = 2.1 Hz, 2 ^1^H, 2′-C*H* + 6′-C*H*), 6.41 (t, *J* = 2.1 Hz, 1 ^1^H, 4′-C*H*), 6.12 (bs, 1 ^1^H, CH_2_N*H*), 3.92 (q, *J* = 6.9 Hz, 2 ^1^H, C*H*_2_CH_3_), 3.72 (s, 6 ^1^H, 2 × OC*H*_3_), 3.74–3.68 (m, 1 ^1^H, 4-C*H*), 3.45 (d, *J* = 10.2 Hz, 1 ^1^H, 2-CH*H*), 3.39 (dd; *J* = 9.6, 7.5 Hz; 1 ^1^H, 5-CH*H*), 3.24–3.18 (m, 1 ^1^H, 5-C*H*H), 3.17 (dd; *J* = 12.9, 8.1 Hz; 1 ^1^H, CH*H*NH), 2.86 (d, *J* = 10.2 Hz, 1 ^1^H, 2-C*H*H), 2.64 (s, 3 ^1^H, NC*H*_3_), 2.59 (dd; *J* = 12.9, 2.1 Hz; 1 ^1^H, C*H*HNH), 1.28 (t, *J* = 6.9 Hz, 1 ^1^H, CH_2_C*H*_3_). ^13^C-NMR [75 MHz, δ (ppm), CDCl3]: 176.7 (*C*O_2_), 160.2 (3′-*C* + 5′-*C*), 155.6 (N*C*ON), 152.9 (4′′-*C*), 139.9 (1′-*C*), 133.9 (1′′-*C*), 119.0 (2′′-*C* + 6′′-*C*), 114.4 (3′′-*C* + 5′′-*C*), 107.1 (2′-*C* + 6′-*C*), 98.3 (4′-*C*), 63.0 (*C*H_2_CH_3_), 62.2 (2-*C*), 59.1 (5-*C*), 55.8 (3-*C*), 55.1 (2 × O*C*H_3_), 49.8 (4-*C*), 42.6 (N*C*H_3_), 40.8 (*C*H_2_NH), 14.7 (CH_2_*C*H_3_). FTIR [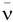 (cm^–1^), neat]: 3354, 3250, 2947, 2836, 1671, 1594, 1542, 1204, 1153, 824. MS [APCI (m/z)] calcd for (C_24_H_31_N_3_O_6_ + H)^+^ = 458, found 458. Crude yield: 52%. Purity: 99% (LC).

*(±)-(3R,4S)-3-{[3-(3-Cyanophenyl)ureido]* *methyl}-4-(3,5-dimethoxyphenyl)-1-methylpyrrolidine-3-*

*carboxylic acid* (**14**{*3*,*7*}): (From **6**{*3*}) ^1^H-NMR [300 MHz, δ (ppm), CD3SOCD3]: 9.88 (bs, 1 ^1^H, N*H*Ar), 7.99 (s, 1 ^1^H, 2′-C*H*), 7.63 (d, *J* = 8.4 Hz, 1 ^1^H, 6′-C*H*), 7.38 (app t, *J* = 7.8 Hz, 1 ^1^H, 5′-C*H*), 7.28 (d, *J* = 7.5 Hz, 1 ^1^H, 4′-C*H*), 6.66 (bs, 1 ^1^H, CH_2_N*H*), 6.47 (d, *J* = 2.1 Hz, 2 ^1^H, 2′′-C*H* + 6′′-C*H*), 6.40 (t, *J* = 2.1 Hz, 1 ^1^H, 4′′-C*H*), 3.77 (app t, *J* = 8.7 Hz, 1 ^1^H, 4-C*H*), 3.71 (s, 6 ^1^H, 2 × OC*H*_3_), 3.59 (d, *J* = 10.5 Hz, 1 ^1^H, 2-CH*H*), 3.54 (dd; *J* = 9.9, 7.5 Hz; 1 ^1^H, 5-CH*H*), 3.37 (app t, *J* = 9.0 Hz, 1 ^1^H, 5-C*H*H), 3.23 (dd; *J* = 12.8, 8.4 Hz; 1 ^1^H, CH*H*NH), 2.97 (d, *J* = 10.5 Hz, 1 ^1^H, 2-C*H*H), 2.75 (s, 3 ^1^H, NC*H*_3_), 2.63 (dd; *J* = 12.8, 1.8 Hz; 1 ^1^H, C*H*HNH). ^13^C-NMR [75 MHz, δ (ppm), CD3SOCD3]: 176.7 (*C*O_2_), 160.2 (3′′-*C* + 5′′-*C*), 155.4 (N*C*ON), 141.8 (1′-*C*), 139.3 (1′′-*C*), 129.9 (5′-*C*), 124.0 (4′-*C*), 122.0 (6′-*C*), 119.8 (2′-*C*), 119.1 (*C*N), 111.3 (3′-*C*), 107.0 (2′′-*C* + 6′′-*C*), 98.4 (4′′-*C*), 62.3 (2-*C*), 58.6 (5-*C*), 55.9 (3-*C*), 55.1 (2 × O*C*H_3_), 49.8 (4-*C*), 42.4 (N*C*H_3_), 40.7 (*C*H_2_NH). FTIR [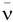 (cm^–1^), neat]: 3377, 3184, 2942, 2838, 2790, 2234, 1679, 1594, 1543, 1203, 1150, 830. MS [APCI (m/z)] calcd for (C_23_H_26_N_4_O_5_ + H)^+^ = 439, found 439. Crude yield: 58%. Purity: >99% (LC).

*(±)-(3R,4S)-4-(3,5-Dimethoxyphenyl)-1-methyl-3-{[3-(3-pyridyl)ureido]* *methyl}pyrrolidine-3-car-*

*boxylic acid* (**14**{*3*,*8*}): (From **6**{*3*}) MS [APCI (m/z)] calcd for (C_21_H_26_N_4_O_5_ + H)^+^ = 415, found 415. Crude yield: 48%. Purity: 43% (LC).

*(±)-(3R,4S)-4-(1,3-Benzodioxol-5-yl)-1-methyl-3-[(3-phenylureido)methyl]pyrrolidine-3-carboxylic*

*acid* (**14**{*4*,*4*}): (From **6**{*4*}) ^1^H-NMR [300 MHz, δ (ppm), CD3SOCD3]: 9.01 (bs, 1 ^1^H, N*H*Ph), 7.42–7.34 (m, 2 ^1^H, 2′′-C*H* + 6′′-C*H*), 7.22–7.12 (m, 2 ^1^H, 3′′-C*H* + 5′′-C*H*), 6.96 (d, *J* = 1.2 Hz, 1 ^1^H, 4′-C*H*), 6.86 (d, *J* = 8.1 Hz, 1 ^1^H, 7′-C*H*), 6.87–6.80 (m, 1 ^1^H, 4′′-C*H*), 6.76 (dd; *J* = 8.1, 1.2 Hz; 1 ^1^H, 6′-C*H*), 6.26 (bd, *J* = 6.0 Hz, 1 ^1^H, CH_2_N*H*), 6.01–5.97 (m, 2 ^1^H, 2′-C*H*_2_), 3.72 (app t, *J* = 8.2 Hz, 1 ^1^H, 4-C*H*), 3.46 (d, *J* = 10.5 Hz, 1 ^1^H, 2-CH*H*), 3.42 (dd; *J* = 9.9, 7.5 Hz; 1 ^1^H, 5-CH*H*), 3.25–3.17 (m, 1 ^1^H, 5-C*H*H), 3.16 (dd; *J* = 12.9, 8.4 Hz; 1 ^1^H, CH*H*NH), 2.88 (d, *J* = 10.5 Hz, 1 ^1^H, 2-C*H*H), 2.65 (s, 3 ^1^H, NC*H*_3_), 2.58 (dd; *J* = 12.9, 2.4 Hz; 1 ^1^H, C*H*HNH). ^13^C-NMR [75 MHz, δ (ppm), CD3SOCD3]: 176.7 (*C*O_2_), 155.4 (N*C*ON), 147.1 (3′a-*C*), 146.1 (7′a-*C*), 140.8 (1′′-*C*), 131.3 (5′-*C*), 128.5 (3′′-*C* + 5′′-*C*), 121.9 (6′-*C*), 120.7 (4′′-*C*), 117.4 (2′′-*C* + 6′′-*C*), 108.9 (7′-*C*), 107.9 (4′-*C*), 100.9 (2′-*C*), 62.2 (2-*C*), 59.4 (5-*C*), 55.9 (3-*C*), 49.5 (4-*C*), 42.8 (N*C*H_3_), 40.5 (*C*H_2_NH). FTIR [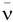 (cm^–1^), neat]: 3359, 3254, 2954, 2898, 2780, 1671, 1597, 1549, 1495, 1228, 1035, 931, 756, 694. MS [APCI (m/z)] calcd for (C_21_H_23_N_3_O_5_ + H)^+^ = 398, found 398. Crude yield: 74%. Purity: >99% (LC).

*(±)-(3R,4S)-4-(1,3-Benzodioxol-5-yl)-1-methyl-3-({3-[4-(trifluoromethyl)phenyl] ureido}methyl)pyrrol-idine-3-carboxylic acid* (**14**{*4*,*5*}): (From **6**{*4*}) FTIR [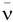 (cm^–1^), neat]: 3358, 3254, 2958, 2904, 1675, 1601, 1546, 1504, 1489, 1321, 1231, 1035, 931. MS [APCI (m/z)] calcd for (C_22_H_22_F_3_N_3_O_5_ + H)^+^ = 466, found 466. Crude yield: 68%. Purity: >99% (LC).

*(±)-(3R,4S)-4-(1,3-Benzodioxol-5-yl)-3-{[3-(4-Ethoxyphenyl)ureido]* *methyl}-1-methylpyrrolidine-3-*

*carboxylic acid* (**14**{*4*,*6*}): (From **6**{*4*}) MS [APCI (m/z)] calcd for (C_23_H_27_N_3_O_6_ + H)^+^ = 442, found 442. Crude yield: 61%. Purity: 77% (LC).

*(±)-(3R,4S)-4-(1,3-Benzodioxol-5-yl)-3-{[3-(3-Cyanophenyl)ureido]* *methyl}-1-methylpyrrolidine-3-*

*carboxylic acid* (**14**{*4*,*7*}): (From **6**{*4*}) MS [APCI (m/z)] calcd for (C_22_H_22_N_4_O_5_ + H)^+^ = 423, found 423. Crude yield: 62%. Purity: 99% (LC).

*(±)-(3R,4S)-4-(1,3-Benzodioxol-5-yl)-1-methyl-3-{[3-(3-pyridyl)ureido]* *methyl}pyrrolidine-3-car-*

*boxylic acid* (**14**{*4*,*8*}): (From **6**{*4*}) MS [APCI (m/z)] calcd for (C_20_H_22_N_4_O_5_ + H)^+^ = 399, found 399. Crude yield: 54%. Purity: 71% (LC).

*(±)-(3R,4S)-1-Methyl-4-(1-methyl-1H-pyrrol-2-yl)-3-[(3-phenylureido)methyl]pyrrolidine-3-*

*carboxylic acid* (**14**{*5*,*4*}): (From **6**{*5*}) MS [APCI (m/z)] calcd for (C_19_H_24_N_4_O_3_ + H)^+^ = 357, found 357. Crude yield: 58%. Purity: 14% (LC).

*(±)-(3R,4S)-1-Methyl-4-(1-methyl-1H-pyrrol-2-yl)-3-({3-[4-(trifluoromethyl)phenyl]* *ureido}methyl)-*

*pyrrolidine-3-carboxylic acid* (**14**{*5*,*5*}): (From **6**{*5*}) MS [APCI (m/z)] calcd for (C_20_H_23_F_3_N_4_O_3_ + H)^+^ = 425, found 425. Crude yield: 65%. Purity: 79% (LC).

*(±)-(3R,4S)-3-{[3-(4-Ethoxyphenyl)ureido]* *methyl}-1-methyl-4-(1-methyl-1H-pyrrol-2-yl)pyrrolidine-*

*3-carboxylic acid* (**14**{*5*,*6*}): (From **6**{*5*}) MS [APCI (m/z)] calcd for (C_21_H_28_N_4_O_4_ + H)^+^ = 401, found 401. Crude yield: 62%. Purity: 16% (LC).

*(±)-(3R,4S)-3-{[3-(3-Cyanophenyl)ureido]methyl}-1-methyl-4-(1-methyl-1H-pyrrol-2-yl)pyrrolidine-3-carboxylic acid* (**14**{*5*,*7*}): (From **6**{*5*}) MS [APCI (m/z)] calcd for (C_20_H_23_N_5_O_3_ + H)^+^ = 382, found 382. Crude yield: 61%. Purity: 99% (LC).

*(±)-(3R,4S)-1-Methyl-4-(1-methyl-1H-indol-3-yl)-3-[(3-phenylureido)methyl]pyrrolidine-3-carboxylic acid* (**14**{*6*,*4*}): (From **6**{*6*}) MS [APCI (m/z)] calcd for (C_23_H_26_N_4_O_3_ + H)^+^ = 407, found 407. Crude yield: 79%. Purity: 97% (LC).

*(±)-(3R,4S)-1-Methyl-4-(1-methyl-1H-indol-3-yl)-3-({3-[4-(trifluoromethyl)phenyl]ureido}methyl)-pyrrolidine-3-carboxylic acid* (**14**{*6*,*5*}): (From **6**{*6*}) MS [APCI (m/z)] calcd for (C_24_H_25_F_3_N_4_O_3_ + H)^+^ = 475, found 475. Crude yield: 76%. Purity: >99% (LC).

*(±)-(3R,4S)-3-{[3-(4-Ethoxyphenyl)ureido]methyl}-1-methyl-4-(1-methyl-1H-indol-3-yl)pyrrolidine-3-carboxylic acid* (**14**{*6*,*6*}): (From **6**{*6*}) ^1^H-NMR [400 MHz, δ (ppm), CD3SOCD3]: 8.92 (bs, 1 ^1^H, N*H*Ar), 7.62 (d, *J* = 7.8 Hz, 1 ^1^H, 4′′-C*H*), 7.38 (d, *J* = 8.4 Hz, 1 ^1^H, 7′′-C*H*), 7.34 (s, 1 ^1^H, 2′′-C*H*), 7.29–7.21 (m, 2 ^1^H, 2′-C*H* + 6′-C*H*), 7.12 (app t, *J* = 7.5 Hz, 1 ^1^H, 6′′-C*H*), 6.96 (app t, *J* = 7.5 Hz, 1 ^1^H, 5′′-C*H*), 6.77–6.69 (m, 2 ^1^H, 3′-C*H* + 5′-C*H*), 6.18 (bd, *J* = 6.9 Hz, 1 ^1^H, CH_2_N*H*), 4.11 (dd; *J* = 9.6, 7.5 Hz; 1 ^1^H, 4-C*H*), 3.91 (q, *J* = 6.9 Hz, 2 ^1^H, C*H*_2_CH_3_), 3.77 (s, 3 ^1^H, 1′′-NC*H*_3_), 3.54 (d, *J* = 10.2 Hz, 1 ^1^H, 2-CH*H*), 3.53–3.49 (m, 1 ^1^H, 5-CH*H*), 3.21 (dd; *J* = 13.2, 8.4 Hz; 1 ^1^H, CH*H*NH), 3.22–3.16 (m, 1 ^1^H, 5-C*H*H), 2.86 (d, *J* = 10.2 Hz, 1 ^1^H, 2-C*H*H), 2.68 (s, 3 ^1^H, 1-NC*H*_3_), 2.66 (dd; *J* = 13.2, 2.1 Hz; 1 ^1^H, C*H*HNH), 1.27 (t, *J* = 6.9 Hz, 3 ^1^H, CH_2_C*H*_3_). FTIR [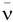 (cm^–1^), neat]: 3330, 2938, 2880, 1664, 1595, 1540, 827, 733. MS [APCI (m/z)] calcd for (C_25_H_30_N_4_O_4_ + H)^+^ = 451, found 451. Crude yield: 70%. Purity: 94% (LC).

*(±)-(3R,4S)-3-{[3-(3-Cyanophenyl)ureido]methyl}-1-methyl-4-(1-methyl-1H-indol-3-yl)pyrrolidine-3-carboxylic acid* (**14**{*6*,*7*}): (From **6**{*6*}) ^1^H-NMR [400 MHz, δ (ppm), CD3SOCD3]: 10.07 (bs, 1 ^1^H, N*H*Ar), 8.02 (s, 1 ^1^H, 2′-C*H*), 7.67 (d, *J* = 8.1 Hz, 1 ^1^H, 6′-C*H*), 7.61 (d, *J* = 7.8 Hz, 1 ^1^H, 4′′-C*H*), 7.39–7.36 (m, 1 ^1^H, 7′′-C*H*), 7.37 (app t, *J* = 7.8 Hz, 1 ^1^H, 5′-C*H*), 7.36 (s, 1 ^1^H, 2′′-C*H*), 7.27 (d, *J* = 7.5 Hz, 1 ^1^H, 4′-C*H*), 7.10 (app t, *J* = 7.5 Hz, 1 ^1^H, 6′′-C*H*), 6.88 (app t, *J* = 7.5 Hz, 1 ^1^H, 5′′-C*H*), 6.74 (bs, 1 ^1^H, CH_2_N*H*), 4.17 (dd; *J* = 9.9, 7.8 Hz; 1 ^1^H, 4-C*H*), 3.77 (s, 3 ^1^H, 1′′-NC*H*_3_), 3.73–3.63 (m, 2 ^1^H, 2-CH*H* + 5-CH*H*), 3.36 (app t, *J* = 9.9 Hz, 1 ^1^H, 5-C*H*H), 3.29 (dd; *J* = 13.4, 9.0 Hz; 1 ^1^H, CH*H*NH), 2.99 (d, *J* = 9.9 Hz, 1 ^1^H, 2-C*H*H), 2.80 (s, 3 ^1^H, 1-NC*H*_3_), 2.68 (dd; *J* = 13.4, 2.1 Hz; 1 ^1^H, C*H*HNH). ^13^C-NMR [75 MHz, δ (ppm), CD3SOCD3]: 177.3 (*C*O_2_), 155.5 (N*C*ON), 142.0 (1′-*C*), 136.6 (7′′a-*C*), 129.8 (5′-*C*), 127.7 (3′′a-*C*), 127.6 (2′′-*C*), 123.9 (4′-*C*), 122.0 (6′-*C*), 121.3 (6′′-*C*), 119.8 (2′-*C*), 119.4 (5′′-*C*), 119.1 (*C*N), 118.6 (4′′-*C*), 111.3 (3′-*C*), 109.6 (3′′-*C*), 109.3 (7′′-*C*), 62.6 (2-*C*), 59.1 (5-*C*), 55.4 (3-*C*), 42.4 (1-N*C*H_3_), 41.9 (4-*C*), 41.0 (*C*H_2_NH), 32.5 (1′′-N*C*H_3_). FTIR [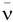 (cm^–1^), neat]: 3361, 2940, 2226, 1685, 1583, 1564, 742. MS [APCI (m/z)] calcd for (C_24_H_25_N_5_O_3_ + H)^+^ = 432, found 432. Crude yield: 67%. Purity: 99% (LC).

*(±)-(3R,4S)-1-Methyl-4-(1-methyl-1H-indol-3-yl)-3-{[3-(3-pyridyl)ureido]methyl}pyrrolidine-3-car-boxylic acid* (**14**{*6*,*8*}): (From **6**{*6*}) MS [APCI (m/z)] calcd for (C_22_H_25_N_5_O_3_ + H)^+^ = 408, found 408. Crude yield: 71%. Purity: 36% (LC).

*(±)-Methyl (3R,4S)-1-methyl-4-phenyl-3-[(3-phenylureido)methyl]pyrrolidine-3-carboxylate* (**15**{*1*,*4*}): Phenyl isocyanate (2.563 g, 21.52 mmol) was added to a solution of α-aminomethyl ester **6**{*1*} (4.858 g, 19.56 mmol) in dry CH_2_Cl_2_ (40 mL). The resulting reaction mixture was stirred at room temperature for 2.5 h and the solvent was then evaporated to afford **15**{*1*,*4*} (6.895 g, 96%) as a white foam, after column chromatography (CH_2_Cl_2_/MeOH, 14:1). ^1^H-NMR [300 MHz, δ (ppm), CDCl3]: 7.47 (bs, 1 ^1^H, N*H*Ph), 7.28–7.15 (m, 9 ^1^H, Ph′ + 2′′-C*H* + 3′′-C*H* + 5′′-C*H* + 6′′-C*H*), 7.03–6.96 (m, 1 ^1^H, 4′′-C*H*), 5.35 (bdd; *J* = 7.8, 3.6 Hz; 1 ^1^H, CH_2_N*H*), 3.91 (app t, *J* = 8.2 Hz, 1 ^1^H, 4-C*H*), 3.70 (s, 3 ^1^H, OC*H*_3_), 3.44 (dd; *J* = 14.1, 8.4 Hz; 1 ^1^H, CH*H*NH), 3.20 (d, *J* = 9.6 Hz, 1 ^1^H, 2-CH*H*), 3.05 (app t, *J* = 8.6 Hz, 1 ^1^H, 5-CH*H*), 2.96 (app t, *J* = 9.0 Hz, 1 ^1^H, 5-C*H*H), 2.80 (dd; *J* = 14.1, 3.9 Hz; 1 ^1^H, C*H*HNH), 2.72 (d, *J* = 9.6 Hz, 1 ^1^H, 2-C*H*H), 2.39 (s, 3 ^1^H, NC*H*_3_).^ 13^C-NMR [75 MHz, δ (ppm), CDCl3]: 176.0 (*C*O_2_), 156.1 (N*C*ON), 138.8 (1′′-*C*), 137.3 (1′-*C*), 129.0 (2′-*C* + 6′-*C*)*, 128.5 (3′-*C* + 5′-*C*)*, 128.4 (3′′-*C* + 5′′-*C*)*, 127.2 (4′-*C*), 123.2 (4′′-*C*), 120.5 (2′′-*C* + 6′′-*C*), 63.3 (2-*C*), 60.4 (5-*C*), 57.0 (3-*C*), 52.8 (O*C*H_3_), 50.8 (4-*C*), 44.0 (*C*H_2_NH), 42.3 (N*C*H_3_). FTIR [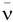 (cm^–1^), neat]: 3323, 2952, 2836, 2790, 1725, 1656, 1597, 1554, 751, 701. HRMS [ESI (m/z)] calcd for (C_21_H_25_N_3_O_3_ + H)^+^ = 368.19687, found 368.19803 (|Δ| = 1.7 ppm). *R*_F_: 0.40 (CH_2_Cl_2_/MeOH, 8:1). Mp: 160.6 °C.

*(±)-(4R,5S)-2-Methyl-4,7-diphenyl-2,7,9-triazaspiro[4,5]decane-6,8-dione* (**5**{*1*,*4*}): A 1 M solution of KOBu*^t^* in THF (120 μL, 120 μmol) was added to a solution of α-ureido ester **15**{*1*,*4*} (44 mg, 120 μmol) in THF (4 mL). The resulting reaction mixture was stirred for 55 min at 31 °C. Brine (1 mL) was then added. The layers were separated and the aqueous phase was extracted with CH_2_Cl_2_ (2 × 1.5 mL). The combined organic layers were dried over Na_2_SO_4_ and concentrated *in vacuo*. The residue afforded **5**{*1*,*4*} (23 mg, 57%) as a white solid, after column chromatography (CH_2_Cl_2_/MeOH, 19:1). ^1^H-NMR [300 MHz, δ (ppm), CD3SOCD3]: 7.79 (bd, *J* = 3.0 Hz, 1 ^1^H, N*H*), 7.43–7.20 (m, 8 ^1^H, Ph′ + 3′′-C*H* + 4′′-C*H* + 5′′-C*H*), 7.16–7.11 (m, 2 ^1^H, 2′′-C*H* + 6′′-C*H*), 4.05 (app t, *J* = 6.9 Hz, 1 ^1^H, 4-C*H*), 2.98 (dd; *J* = 9.0, 6.6 Hz; 1 ^1^H, 3-CH*H*), 2.95–2.84 (m, 4 ^1^H, 1-C*H*_2_ + 3-C*H*H + 10-CH*H*), 2.67 (dd; *J* = 12.6, 1.5 Hz; 1 ^1^H, 10-C*H*H), 2.36 (s, 3 ^1^H, NC*H*_3_). ^13^C-NMR [75 MHz, δ (ppm), CDCl3]: 173.3 (6-*C*), 153.9 (8-*C*), 139.0 (1′-*C*), 135.4 (1′′-*C*), 129.1 (3′′-*C* + 5′′-*C*), 128.63 (3′-*C* + 5′-*C*)*, 128.58 (2′′-*C* + 6′′-*C*)*, 128.5 (2′-*C* + 6′-*C*)*, 128.4 (4′′-*C*), 127.2 (4′-*C*), 64.0 (1-*C*), 62.0 (3-*C*), 52.5 (5-*C*), 48.3 (4-*C*), 43.7 (10-*C*), 42.1 (N*C*H_3_). FTIR [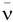 (cm^–1^), neat]: 3254, 2945, 2835, 2792, 1725, 1684, 767, 753, 707, 693. Elem. anal. calcd for C_20_H_21_N_3_O_2_: C 71.62%, H 6.31%, N 12.53%; found C 71.70%, H 6.17%, N 12.48%. *R*_F_: 0.44 (CH_2_Cl_2_/MeOH, 8:1). Mp: 231.5 °C (from MeOH, colorless crystals). Purity: >99.5% (GC).

## 5. Conclusions

In summary, we have developed a general high-yielding method for the synthesis of *trans*-4-aryl-substituted 3-(aminomethyl)pyrrolidine-3-carboxylates. We have shown that these aryl groups can be phenyl or electron-rich aryls (4-methoxyphenyl, 3,5-dimethoxyphenyl, and 1,3-benzodioxol-5-yl) and electron-rich heteroaryls (1-methylpyrrol-2-yl and 1-methylindol-3-yl). As a result, we anticipate that this methodology can be successfully applied for a wide range of aromatic groups, although with electron-poor heteroaryls (2-, 3-, and 4-pyridyl) mixtures of *cis*–*trans* isomers are formed after the cycloaddition step.

We have also developed a method for the synthesis of a small library of 7-alkyldihydrouracils spiro-fused to pyrrolidines to the 3-position. The corresponding 7-aryl derivatives hydrolyzed under the conditions utilized for the cyclization and yielded α-ureidomethyl acids. The optimization of this cyclization should be further developed.
